# Effectiveness and Safety of Liuwei Dihuang as an Adjunctive Therapy for Cognitive Impairment: A Systematic Review, Meta-Analysis, and Network Pharmacology Analysis

**DOI:** 10.3390/ph19050776

**Published:** 2026-05-15

**Authors:** Jihyun Hwang, Mi Hye Kim, Jeongrim Bak, Jong-Min Yun, Jungtae Leem

**Affiliations:** 1 Department of Korean Internal Medicine, College of Korean Medicine, Wonkwang University, 460, Iksan-daero, Iksan-si 54538, Jeollabuk-do, Republic of Korea; con2858@naver.com (J.H.); gogogreenms@gmail.com (J.B.); 2 College of Korean Medicine, Woosuk University, 61 Seonneomeo 3-gil, Wansan-gu, Jeonju-si 55338, Jeonbuk-do, Republic of Korea; kimmh526@woosuk.ac.kr; 3 Research Center of Traditional Korean Medicine, College of Korean Medicine, Wonkwang University, 460, Iksan-daero, Iksan-si 54538, Jeollabuk-do, Republic of Korea; 4 Department of Diagnostics, College of Korean Medicine, Wonkwang University, 460, Iksan-daero, Iksan-si 54538, Jeollabuk-do, Republic of Korea; 5 Department of Integrated Medicine, Wonkwang University Korean Medicine Hospital, 895, Muwang-ro, Iksan-si 54538, Jeollabuk-do, Republic of Korea

**Keywords:** cognitive impairment, Liuwei Dihuang, systematic review, meta-analysis, network pharmacology, herbal medicine, network cluster

## Abstract

**Background/Objectives**: Liuwei Dihuang (LWDH) is a classical plant-derived herbal formula widely used for cognitive decline. This study aimed to evaluate its efficacy and safety in cognitive disorders and to explore its potential pharmacological mechanisms using network pharmacology. **Methods**: We searched 11 databases through November 2024 for randomized controlled trials comparing LWDH plus conventional therapy with conventional therapy alone in cognitive disorders. Meta-analysis was performed for clinical outcomes, and herb–compound–target and disease-target datasets were integrated to identify core molecular modules. **Results**: Twelve randomized controlled trials involving 1137 participants were included. Adjunctive LWDH was associated with improvements in Mini-Mental State Examination scores (MD = 2.34, 95% CI 0.88–3.79), activities of daily living, and quality of life. However, substantial heterogeneity and methodological limitations, including unclear randomization and blinding, were observed across studies, indicating a potential risk of bias. Fewer adverse events were reported in the LWDH plus conventional treatment group, although reporting quality was limited. The overall risk of bias was judged as “some concerns”. Network pharmacology analysis identified a broad set of overlapping genes between LWDH-associated targets and cognitive disorder-related genes, which were further refined through filtering procedures. Subsequent analyses suggested associations with pathways related to neurodegeneration, apoptosis, and central nervous system function; however, these findings are exploratory and based on in silico predictions. **Conclusions**: LWDH may be associated with potential adjunctive benefits in cognitive disorders. However, given the methodological limitations and clinical heterogeneity of the included studies, the findings should be interpreted with caution. The proposed pharmacological mechanisms are exploratory and require further validation. Well-designed randomized controlled trials are needed to establish more robust evidence.

## 1. Introduction

The Diagnostic and Statistical Manual of Mental Disorders (DSM-5) defines neurocognitive disorders (NCDs) as acquired cognitive impairments caused by various conditions across all age groups. NCDs are classified into “major” and “mild” forms based on the severity of cognitive and functional impairment [1]. Dementia corresponds to major NCD and is characterized by progressive decline in memory, cognition, and daily functioning. Alzheimer’s disease is the most common cause, accounting for approximately 60–70% of cases, followed by vascular, Lewy body, and frontotemporal dementia [2].

The global burden of dementia has increased rapidly with population aging. According to the World Health Organization, approximately 55 million people worldwide were living with dementia in 2021, and this number is expected to rise to 139 million by 2050 [3]. Dementia imposes substantial economic and social burdens, with global costs exceeding $1 trillion annually [3,4].

Current pharmacological treatments, including cholinesterase inhibitors (donepezil, rivastigmine, and galantamine) and the N-methyl-D-aspartate (NMDA) receptor antagonist memantine, are primarily aimed at symptomatic management rather than disease modification [5]. While these treatments may provide modest benefits, their efficacy varies across dementia subtypes and disease stages [6]. In addition, dementia is frequently accompanied by behavioral and psychological symptoms, such as sleep disturbances, depression, agitation, and hallucinations, which often require additional pharmacological management [7].

While pharmacological treatments can alleviate symptoms, they often present side effects such as sedation and agitation, emphasizing the need for complementary non-pharmacological interventions. Preventive strategies include cardiovascular risk management, regular physical activity, cognitive training, and cognitive stimulation therapy. Alternative therapies such as aromatherapy, music therapy, massage, and light therapy have been explored for managing behavioral and psychological symptoms, though their effectiveness remains uncertain [8,9].

In East Asia, traditional herbal medicines (HMs) are frequently used alongside conventional treatments for dementia. Several systematic literature reviews have explored the role of HMs [10], supporting their consideration as a complementary treatment approach. Liuwei Dihuang (LWDH) has traditionally been employed to address conditions associated with kidney yin deficiency, often linked to aging. This prescription consists of six herbal ingredients: *Rehmannia glutinosa* (Shu Di Huang), *Dioscorea batatas* (Shan Yao), *Cornus officinalis* (Shan Zhu Yu), *Paeonia suffruticosa* (Mu Dan Pi), *Alisma orientale* (Ze Xie), and *Poria cocos* (Fu Ling) [11,12].

Recent animal studies have demonstrated its pharmacological effects on renal function, immune regulation, blood sugar control, and conditions such as diabetes, hypertension, hyperlipidemia, and cardiovascular disease [13,14,15,16,17,18]. In addition, LWDH has shown potential effects in mitigating age-related cognitive decline, improving cognitive impairment associated with cholinergic dysfunction, and attenuating dementia-related changes in preclinical models [19,20,21,22]. It has also been reported to reduce oxidative stress and inflammation in the hippocampus and to improve memory impairment induced by chronic stress [23,24]. Clinical studies have further suggested potential benefits in neurodegenerative conditions, including Parkinson’s disease, with reported improvements in symptoms and quality of life [25].

Network pharmacology studies have explored potential mechanisms of LWDH in Alzheimer’s disease, suggesting its involvement in pathways related to oxidative stress, neuroinflammation, and neuronal survival [26,27]. However, because herbal formulae are multi-component and multi-target interventions, identifying precise molecular mechanisms remains challenging.

Despite accumulating clinical and experimental evidence supporting the potential of LWDH in treating cognitive impairment, definitive conclusions regarding its efficacy and safety remain elusive. A previous meta-analysis on LWDH for Alzheimer’s disease [28] suggested favorable effects, but it was limited by its reliance on non-validated outcomes such as total effective rate, insufficient separation of study designs, and lack of a formal certainty-of-evidence assessment. In addition, more recent trials and biomarker-related findings have not been comprehensively integrated.

Since 2019, studies incorporating hematological indicators have been conducted [29,30,31] but these have not yet been fully synthesized in systematic reviews. While systematic reviews and meta-analyses provide robust clinical evidence regarding treatment efficacy and safety [32], they do not fully capture the biological context of multi-component herbal medicines. Because herbal formulations involve multiple compounds acting on diverse targets, clinical evidence alone may not sufficiently explain their potential mechanisms of action [33,34]. Therefore, in this study, network pharmacology was applied as an exploratory and hypothesis-generating approach to provide complementary mechanistic context rather than to validate clinical efficacy.

Therefore, this study aimed to systematically evaluate the efficacy and safety of LWDH for cognitive impairment using a meta-analysis and the GRADE methodology. In addition, network pharmacology was applied as an exploratory approach to provide complementary insights into potential pharmacological mechanisms.

## 2. Results

### 2.1. Description of Included Studies

**Figure 1:**
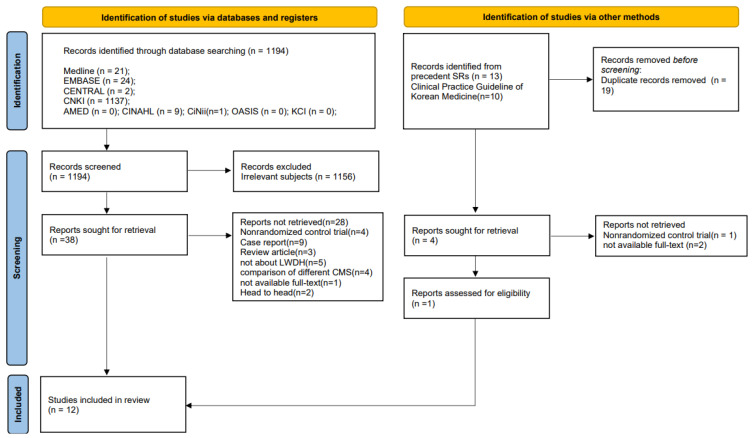
PRISMA flow chart of the study selection process.

A comprehensive database search yielded 1194 studies. Following an initial screening based on titles and abstracts per PRISMA guidelines [35], 39 studies were identified. Twelve studies were included in the meta-analysis (Figure 1) (The excluded studies and reasons for exclusion are detailed in Supplementary Content S2). No automation tools were used for study selection; all exclusions were performed manually by the researchers.

The distribution of studies by year, along with details on the study design, subjects, therapeutic interventions and controls, outcome variables, safety, and follow-up investigations, are described in Table 1.

All the studies were conducted in China. In 11 studies, a comparison was made between a group receiving standard treatment and another receiving standard treatment with LWDH, whereas only one study compared a group receiving a combination of HM and standard treatment with a group receiving a combination of placebo LWDH and standard treatment. The number of participants in the studies ranged from 60 to 120, with a total of 1137 participants. The treatment periods ranged from eight weeks to six months, with three months being the most common duration in five studies, and the average duration was approximately 15.3 weeks.

Various diagnostic criteria and traditional Chinese medicine (TCM) pattern differentiation tools were used across studies. Four studies employed the Diagnostic and Statistical Manual of Mental Disorders (DSM-IV), whereas two used the Guidelines for Clinical Research of New Drugs of Traditional Chinese Medicine and the Chinese Classification of Mental Disorders-3 (CCMD-3). Additional studies referenced other national or consensus-based diagnostic and treatment guidelines for dementia, vascular dementia, or cognitive disorders (Supplementary Content S3).

In the included studies, three types of conventional medications were used as comparators: donepezil hydrochloride in six studies [39,40,41,42,43,44], memantine in three studies [29,31,38], and olanzapine in two studies [36,37].

### 2.2. Methodological Quality

Methodological quality was assessed using version 2 of the Cochrane risk-of-bias tool for randomized trials (RoB 2). Randomization methods were generally inadequately reported. In six studies [30,31,36,40,42,43], the use of a random number table was described; however, because allocation concealment was not reported, these studies were judged as having a “some concerns” risk of bias for the randomization process. One study [38] also reported the use of a random number table, but the description was unclear and was therefore similarly judged as “some concerns.” In five studies [29,37,39,41,44], participants were described as randomized, but no details were provided regarding the randomization method or allocation concealment, resulting in a “some concerns” judgement.

None of the studies reported blinding of participants, caregivers, intervention providers, or outcome assessors. Given the nature of herbal medicine interventions, blinding may have been difficult to implement; however, the lack of reporting raises concerns regarding potential performance and detection bias.

**Figure 2:**
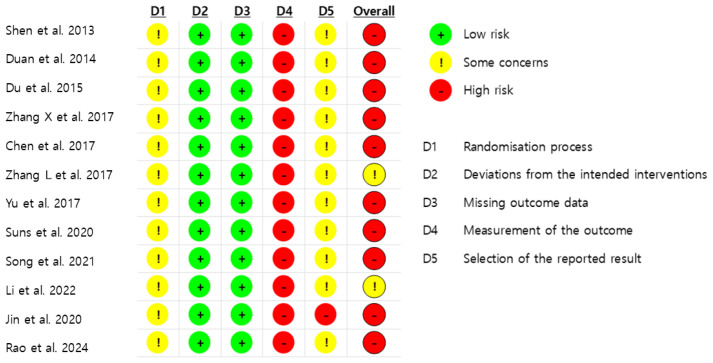
Risk of bias summary and graph for all included studies [29,30,31,36,37,38,39,40,41,42,43,44].

All studies reported complete outcome data for the analyzed samples, and the risk of bias due to missing outcome data was judged as low. Although outcome measures were generally applied consistently, the absence of blinding in outcome assessment may have influenced the evaluation process, resulting in a judgement of high risk of bias in this domain. Selective reporting bias was generally judged as “some concerns” because study protocols were not available in most cases. One study [43] was judged to be at high risk of selective reporting bias because, although multiple outcomes were assessed, only TER was reported as the primary effectiveness outcome (Figure 2).

### 2.3. Details of LWDH Administration

In terms of formulation, three studies used decoctions, whereas the remaining studies employed pill formulations. The frequency of administration was twice daily in eight studies and three times daily in four studies.

Across studies, LWDH was administered based on its standard six-herb composition, including *Rehmannia glutinosa* (Shu Di Huang), *Dioscorea batatas* (Shan Yao), *Cornus officinalis* (Shan Zhu Yu), *Paeonia suffruticosa* (Mu Dan Pi), *Alisma orientale* (Ze Xie), and *Poria cocos* (Fu Ling), which were consistently used in all studies. Additional herbal components were incorporated in some studies, and honey (Feng Mi) was used in four studies (Supplementary Content S4).

### 2.4. Clinical Outcome Variables and Quantitative Synthesis

#### 2.4.1. Clinical Outcome Variables of LWDH Plus WM vs. WM Alone

Eleven studies evaluated the combined effects of LWDH and WM compared to WM alone.

- Cognitive Function

**Figure 3:**
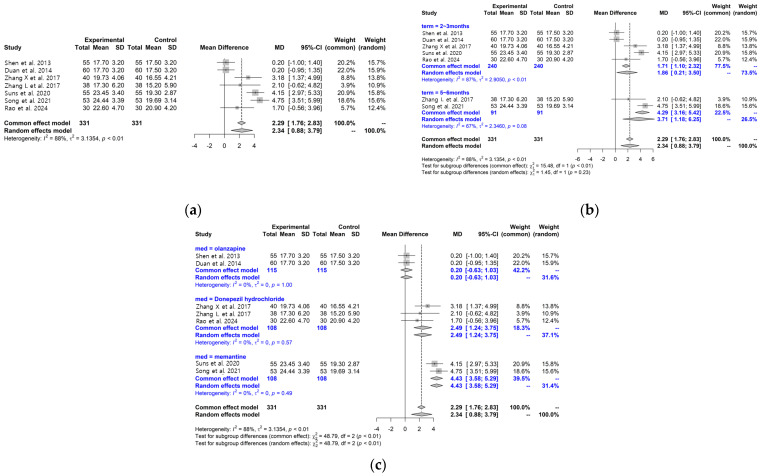
Forest plots for MMSE and subgroup analysis between the LWDH plus conventional treatment group and the conventional treatment alone group (**a**) MMSE; (**b**) MMSE Subgroup analysis according to treatment period; and (**c**) MMSE Subgroup analysis by WM [29,31,36,37,41,42,44].

Seven studies [29,31,36,37,39,41,44] assessed cognitive function using the Mini-Mental State Examination (MMSE). Pooled analysis showed higher MMSE scores in the LWDH plus conventional treatment group compared with the control group; however, substantial heterogeneity was observed (MD: 2.34, 95% CI: 0.88–3.79, I^2^ = 88%), and the pooled estimate should therefore be interpreted cautiously (Figure 3a). Formal meta-regression was not performed for MMSE because only seven studies were available for this outcome, and regression analyses based on such a small number of studies would be statistically unstable and potentially misleading.

Subgroup analyses according to treatment duration showed higher MMSE scores in both the 2–3 month subgroup [29,36,37,41,44] and the 5–6 month subgroup [31,42], although heterogeneity remained. Differences between subgroups were observed (*p* = 0.23), but these findings should be interpreted cautiously given the limited number of studies (Figure 3b).

In subgroup analyses based on comparator medications, studies using olanzapine [36,37] showed no clear difference, whereas studies using donepezil [41,42,44] or memantine [29,31] showed higher MMSE scores in the intervention group. Differences between subgroups were observed (*p* < 0.01); however, these results should be considered exploratory due to the small number of studies within each subgroup (Figure 3c). The observed heterogeneity may be related to differences in dementia subtype, baseline cognitive severity, and comparator treatments.

Two studies [29,42] assessed cognitive function using ADAS-Cog and reported no clear difference between groups (MD: −7.86, 95% CI: −16.96–1.24, I^2^ = 89%). Three studies [38,39,40] used ACE-R and also reported no clear difference (MD: 0.70, 95% CI: −0.02–1.42, I^2^ = 0%) (Supplementary Content S6.1).

- Activities of Daily Living and Quality of Life

**Figure 4:**
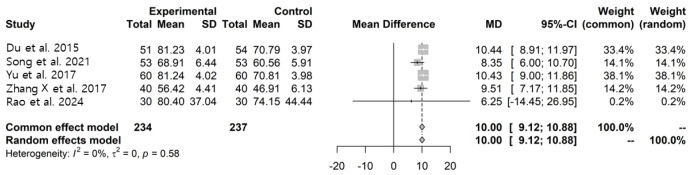
Forest plots for ADL (MBI) between the LWDH plus conventional treatment group and the conventional treatment alone group [31,38,39,41,44].

Five studies [31,38,39,41,44] assessed activities of daily living using the Modified Barthel Index (MBI). Pooled analysis showed higher MBI scores in the LWDH plus conventional treatment group compared with the control group (MD: 10.00, 95% CI: 9.12–10.88, I^2^ = 0%) (Figure 4).

Subgroup analyses according to comparator medication showed similar patterns in studies using memantine and donepezil, although moderate heterogeneity was observed in the memantine subgroup. Two studies [29,42] assessing activities of daily living using alternative scales also reported differences between groups (MD: −2.66, 95% CI: −3.79–−1.53, I^2^ = 0%). Two studies [29,31] evaluating quality of life using QOL-AD showed higher scores in the intervention group; however, substantial heterogeneity was observed (MD: 4.77, 95% CI: 0.48–9.06, I^2^ = 92%) (Supplementary Content S6.2).

- Mental State

Six studies [31,36,37,38,39,40] assessed neuropsychiatric symptoms using BEHAVE-AD. Pooled analysis showed no clear difference between groups (MD: −0.58, 95% CI: −1.80–0.64, I^2^ = 91%) (Supplementary Content S6.3). Subgroup analyses also did not show consistent differences.

- Traditional Chinese Medicine (TCM) symptom score

Two studies [42,44] reported TCM symptom scores. No clear difference between groups was observed (MD: −4.46, 95% CI: −8.96–0.33) (Supplementary Content S6.4).

- Total Effective Rate (TER)

**Figure 5:**
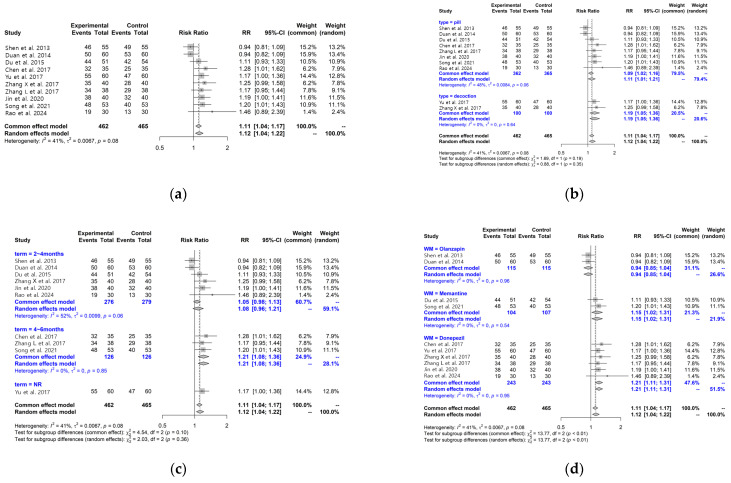
Forest plots for TER and subgroup analysis between the LWDH plus conventional treatment group and the conventional treatment alone group: (**a**) TER; (**b**) TER subgroup analysis according to LWDH type; (**c**) TER subgroup analysis according to treatment period; and (**d**) TER subgroup analysis by WM [31,36,37,38,39,40,41,42,43,44].

Ten studies [31,36,37,38,39,40,41,42,43,44] reported TER based on clinical symptom criteria. Pooled analysis showed higher TER in the LWDH plus conventional treatment group compared with the control group (RR: 1.12, 95% CI: 1.04–1.22, I^2^ = 41%) (Figure 5a).

Subgroup analyses according to formulation, treatment duration, and comparator medications showed variable patterns; however, differences between subgroups were not statistically significant and should be interpreted cautiously (Figure 5b–d). The criteria used to define TER in each study are provided in Supplementary Content S5.

- Hematological Indicators

Two studies reported biomarker outcomes. One study [31] assessed BDNF, Aβ, and IL-1β, and another study [29] assessed oxidative stress markers (MDA, SOD, and GSH-Px). Differences between groups were reported in these studies; however, these findings were derived from a small number of studies and should be interpreted cautiously.

#### 2.4.2. LWDH vs. Placebo

One study [30] compared LWDH with a placebo in a subgroup of hypertensive patients with cognitive impairment. This study reported higher cognitive scores and differences in several biomarkers in the LWDH group compared with placebo. However, as these findings were derived from a single study in a specific population, they should be interpreted cautiously.

### 2.5. Follow-Up

One study [30] conducted a follow-up assessment six months after treatment completion. Cognitive function was evaluated using the MMSE and MoCA. Higher scores were reported in the LWDH plus conventional treatment group compared with the placebo plus conventional treatment group (MMSE: MD = 2.33, 95% CI: 1.10–3.56; MoCA: MD = 2.74, 95% CI: 1.45–4.03). However, as these findings were derived from a single study, they should be interpreted cautiously.

### 2.6. Subgroup Analysis

Subgroup analyses were conducted based on comparator medications, treatment duration, and formulation of LWDH. Overall, most subgroup comparisons did not show statistically significant differences; however, some variations were observed. In subgroup analyses of MMSE according to comparator medication, studies using olanzapine showed no clear difference between groups, whereas studies using donepezil hydrochloride or memantine showed higher scores in the intervention group. Differences between subgroups were observed; however, these findings should be interpreted cautiously given the small number of studies within each subgroup.

In subgroup analyses of TER according to treatment duration, studies with longer treatment durations (5–6 months) showed higher point estimates compared with shorter-duration studies (2–4 months), although these differences were not statistically significant (*p* = 0.23). Subgroup analyses based on comparator medication showed differences between groups, but these results were also based on a limited number of studies.

Subgroup analyses according to formulation (decoction vs. pill) showed similar effects across groups without statistically significant differences. Overall, subgroup findings should be regarded as exploratory because of the small number of studies in each subgroup and the heterogeneity of clinical contexts.

### 2.7. Safety

Among the 12 included studies, seven [29,30,31,36,38,39,40] reported adverse events. Most studies compared LWDH plus conventional treatment with conventional treatment alone, while one study [30] compared LWDH plus conventional treatment with placebo plus conventional treatment. In five studies [29,31,36,38,39], a greater number of adverse events was reported in the conventional treatment group compared with the LWDH plus conventional treatment group, whereas two studies [30,40] reported no adverse events in either group.

**Figure 6:**
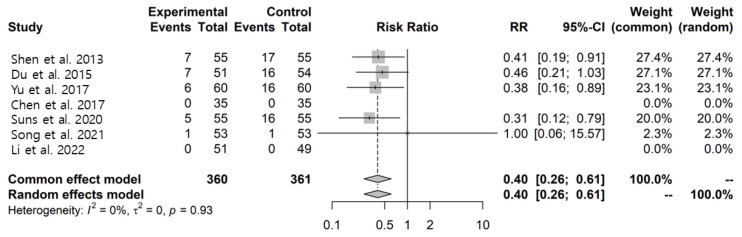
Forest plots for the number of adverse effects and subgroup analysis between the LWDH plus conventional treatment group and the conventional treatment alone group [29,30,31,36,38,39,40].

Across studies, adverse events in the conventional treatment group included dizziness, headache, somnolence, insomnia, extrapyramidal symptoms, and gastrointestinal disorders. Similar types of adverse events were reported in the intervention group, although fewer cases were observed. Pooled analysis suggested fewer reported adverse events in the LWDH plus conventional treatment group compared with the conventional treatment group (RR: 0.40, 95% CI: 0.26–0.61, I^2^ = 0%) (Figure 6). However, given the limited reporting quality and methodological limitations of the included studies, these findings should be interpreted cautiously.

### 2.8. Quality of Evidence

The certainty of evidence for comparisons between LWDH plus conventional treatment and conventional treatment alone ranged from low to moderate according to the GRADE approach. The primary reasons for downgrading included risk of bias due to inadequate reporting of randomization and blinding, as well as imprecision related to small sample sizes and wide confidence intervals (Table 2).

### 2.9. Sensitivity Analysis

Sensitivity analyses were performed to assess the stability of the pooled results. Comparisons between fixed-effects and random-effects models showed similar effect estimates across most outcomes, with overlapping confidence intervals and consistent directions of effect. These findings suggest that the overall results were not substantially influenced by the choice of statistical model. In addition, leave-one-out sensitivity analyses were conducted for key outcomes, including MMSE, ADL, and BEHAVE-AD. Sequential exclusion of individual studies did not materially alter the pooled estimates, indicating that no single study had a disproportionate influence on the overall results (Supplementary Content S7).

### 2.10. Publication Bias

**Figure 7:**
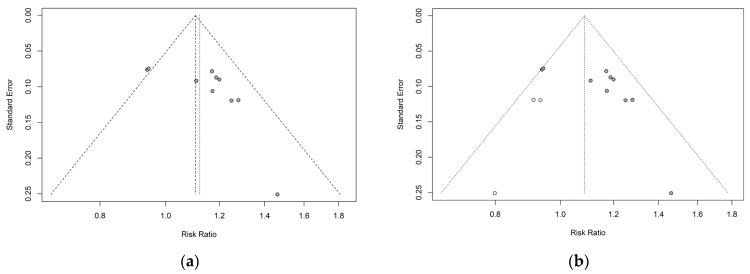
(**a**) Funnel plot showing potential publication bias among included studies before adjustment. (**b**) Funnel plot after applying the trim-and-fill method to account for potentially missing studies. Dots represent individual studies, and the dashed line represents the pooled effect estimate.

Potential publication bias was evaluated using funnel plots and Egger’s test (Figure 7a). Visual inspection of the funnel plot suggested possible asymmetry, which was further supported by Egger’s test (*p* = 0.0426). After applying the trim-and-fill method [45], three studies were imputed; however, the adjusted effect estimates remained largely unchanged (Figure 7b). These findings should be interpreted cautiously, as the assessment of publication bias was limited. However, formal assessment of publication bias was limited because fewer than 10 studies were available for most outcome-specific meta-analyses. Under these conditions, funnel plot interpretation and statistical tests for asymmetry are considered unreliable, and the findings should therefore be interpreted cautiously.

### 2.11. Identification of Putative Targets for LWDH in Cognitive Disorders

**Figure 8:**
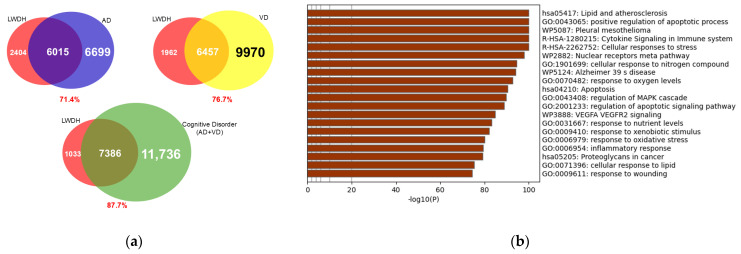
Identification and global functional analysis of putative therapeutic targets of LWDH for cognitive disorders. (**a**) Venn diagram illustrating the intersection of LWDH-associated genes and cognitive disorder-related targets genes including Alzheimer’s disease and vascular dementia. (**b**) Gene Ontology (GO) and Kyoto Encyclopedia of Genes and Genomes (KEGG) pathway enrichment analysis of the overlapping genes.

The potential targets of LWDH in cognitive disorders, including Alzheimer’s disease and vascular dementia, were explored by comparing their respective gene sets. Among the 8419 LWDH-associated genes, 6015 and 6457 genes overlapped with the gene sets of Alzheimer’s disease and vascular dementia, respectively, corresponding to 71.4% and 76.7%. The combined and deduplicated gene set for cognitive disorders consisted of 19,112 genes. A large number of putative overlapping genes were identified between LWDH-associated targets and the cognitive disorder gene set, with 7386 genes (87.7%) overlapping (Figure 8a). This high degree of overlap likely reflects the broad nature of target prediction and does not indicate disease-specific biological relevance.

The overlapping gene set was subsequently subjected to enrichment analysis using multiple databases, including Gene Ontology (GO), Kyoto Encyclopedia of Genes and Genomes (KEGG), Reactome, and WikiPathways. These analyses identified a range of associated biological processes and pathways. Notably, pathways related to Alzheimer’s disease (e.g., WP5124) were among the enriched terms; however, these findings should be interpreted as exploratory functional associations derived from a large candidate gene set (Figure 8b).

### 2.12. Modular Clustering Analysis of the PPI Network

**Figure 9:**
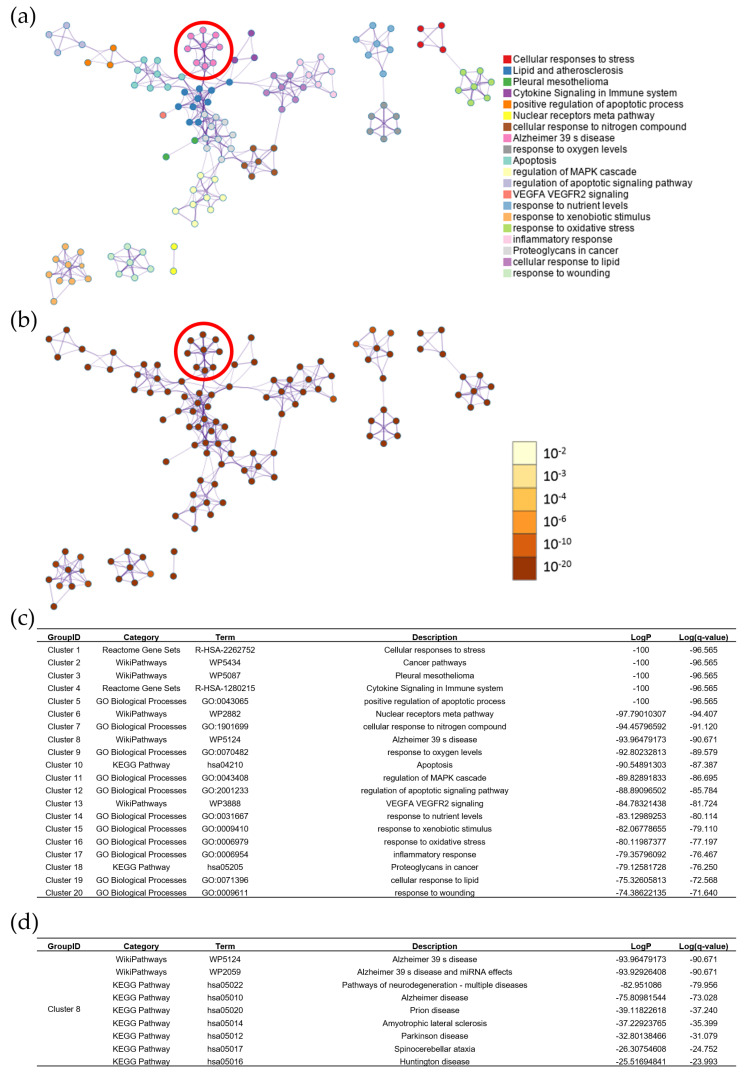
Protein–protein interaction (PPI) network clustering and modular analysis. (**a**) The distinct modules (clusters) of the overlapping genes PPI network color-coded. (**b**) The distribution of statistical significance across the identified clusters with the *p* value. (**c**) Summary of functional enrichment terms associated with each distinct cluster. (**d**) Detailed enrichment analysis of the Cluster 8. The red circles indicate the highlighted Cluster 8 associated with Alzheimer’s disease-related pathways.

To improve interpretability and reduce potential noise from the large overlapping gene set, a subset of 1498 genes with higher disease-relevance scores was selected for protein–protein interaction (PPI) network construction (Figure 9). The PPI network was analyzed using modular clustering to identify functionally related gene groups. Among the identified clusters, Cluster 8 was associated with pathways related to Alzheimer’s disease based on enrichment analysis. This cluster was located in proximity to clusters enriched for apoptosis and lipid- and atherosclerosis-related pathways, suggesting potential functional relationships among these biological processes. Functional enrichment analysis using the Metascape platform (v3.5.20240101; https://metascape.org/), adjusted for multiple testing, identified pathways related to neurodegeneration across multiple databases, including WikiPathways and KEGG. These findings suggest that the identified gene clusters may reflect associations with biological processes related to neurodegeneration; however, they do not establish causal relationships.

### 2.13. Functional Pathway and Tissue-Specific Expression of the Core Module

**Figure 10:**
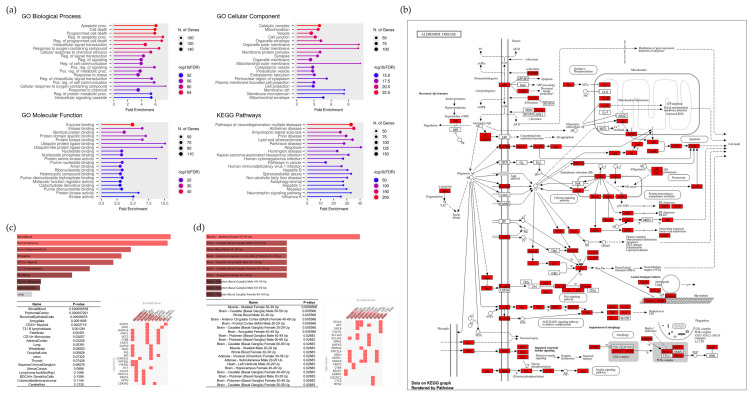
Functional annotation and tissue-specific expression profiling of Cluster 8. (**a**) Functional enrichment analysis of 226 target genes in the Cluster 8 using ShinyGO. (**b**) Schematic diagram of the highly enriched KEGG pathway associated with Cluster 8. (**c**) Tissue-specific expression analysis of Cluster 8 genes based on the Human Gene Atlas. (**d**) Tissue expression profiling demonstrating central nervous system (CNS) specificity based on GTEx Tissue Sample Gene Expression Up data.

To further characterize the functional properties of Cluster 8, enrichment analysis was performed on 226 genes within this module (Figure 10). Gene Ontology (GO) analysis indicated associations with biological processes such as apoptosis, cell death, and programmed cell death. Cellular component analysis suggested enrichment in catalytic complexes, mitochondria, vesicles, and cell junctions, while molecular function analysis identified enzyme binding, kinase binding, and protein binding. KEGG pathway analysis suggested associations with multiple neurodegeneration-related pathways, including Alzheimer’s disease, amyotrophic lateral sclerosis, and prion disease. These results indicate that the identified gene set may be associated with biological processes relevant to neurodegeneration, although such associations remain indirect and exploratory.

Tissue-specific expression analysis was conducted using the Enrichr platform. The Human Gene Atlas database indicated that genes within Cluster 8 were enriched in central nervous system (CNS) tissues, including the prefrontal cortex, amygdala, fetal brain, and whole brain. Similarly, GTEx data suggested enrichment in brain regions associated with cognitive function, such as the caudate nucleus (basal ganglia) and anterior cingulate cortex. While these findings provide supportive biological context, they are based on in silico analyses and do not confirm functional activity or causal mechanisms. Therefore, these results should be interpreted as hypothesis-generating, and further experimental validation is required.

## 3. Discussion

This systematic review and meta-analysis evaluated the effectiveness and safety of Liuwei Dihuang (LWDH) as an adjunctive therapy for cognitive impairment associated with neurodegenerative disorders. The pooled results from 12 randomized controlled trials suggest that LWDH, when used in combination with conventional treatment, may be associated with higher cognitive scores, improved activities of daily living, and better quality of life compared with conventional treatment alone. In addition, fewer adverse events were reported in the combination group. However, these findings should be interpreted with caution. The included studies were limited by methodological weaknesses, including insufficient reporting of randomization procedures, lack of blinding, and relatively small sample sizes, resulting in low to moderate certainty of evidence. Furthermore, substantial clinical heterogeneity was observed across studies in terms of disease types, comparator treatments, intervention characteristics, and treatment duration.

Previous meta-analyses have reported generally favorable effects of LWDH [28]; however, these studies were constrained by methodological limitations, including reliance on non-validated outcomes such as total effective rate (TER), lack of clear separation between study designs, and insufficient assessment of evidence certainty. In contrast, the present study focused on add-on designs and prioritized validated clinical outcomes. Although the overall direction of findings is broadly consistent with previous reports [28], the current analysis emphasizes cautious interpretation in light of the limitations of the available evidence. From a clinical perspective, the findings suggest that LWDH may have potential as an adjunctive therapeutic option. However, the magnitude and consistency of its effects remain uncertain. Although pooled estimates for cognitive outcomes such as MMSE suggest higher scores in the intervention group, their clinical relevance remains uncertain given the substantial heterogeneity and methodological limitations of the included studies. Reported minimal clinically important difference (MCID) thresholds for MMSE in dementia-related conditions have varied across studies, generally falling in the range of approximately 1.4 to 3 points depending on disease severity and the anchor used. In this context, the pooled MMSE difference observed in the present review (MD = 2.34) may fall within the lower bound of a potentially clinically meaningful range. However, given the substantial heterogeneity, mixed clinical populations, and very low certainty of evidence for this outcome, the clinical meaningfulness of this estimate remains uncertain and should be interpreted cautiously [46]. Differences in disease subtype, baseline severity, comparator treatments, and intervention protocols may have contributed to the observed variability. It is also important to note that validated clinical scales such as MMSE, ADL, and QOL-AD provide more reliable outcome measures than non-standardized indices such as TER. Accordingly, TER was treated as a secondary and exploratory outcome in this study and was not used as a primary basis for clinical interpretation. The integration of network pharmacology in this study was intended to provide exploratory biological context for the observed clinical findings. The analysis identified gene clusters associated with biological processes related to neurodegeneration, including Alzheimer’s disease and apoptosis-related pathways [47]. However, these findings should be interpreted as hypothesis-generating rather than confirmatory.

The large number of overlapping genes identified in the initial analysis reflects the broad scope of in silico target prediction and does not necessarily indicate disease-specific biological relevance. To improve interpretability, a filtering step based on disease-relevance scores was applied to reduce noise and minimize false-positive associations inherent in large-scale target prediction datasets [48,49]. Similarly, pathway enrichment results represent statistical associations rather than direct evidence of causal mechanisms. Some experimental studies have reported biological activities of individual components of LWDH, including potential effects on oxidative stress, neuroinflammation, and neuronal function [27,50,51,52]. However, these findings are primarily derived from preclinical models and do not directly validate the mechanisms suggested by the present network pharmacology analysis.

Although tissue-specific expression analysis suggested enrichment in central nervous system regions relevant to cognitive function [53,54], these findings remain indirect and based on computational inference. Therefore, the network pharmacology results should be regarded as complementary to the clinical findings rather than explanatory, and further experimental validation is required. In traditional East Asian medicine, cognitive impairment has been conceptualized in relation to deficiency-related patterns [55]. Some included studies applied such classifications; however, findings were inconsistent, and further research is required to clarify potential differential effects.

This study has several strengths. A comprehensive search strategy was employed across multiple international and regional databases, including East Asian databases, which are particularly relevant for traditional herbal medicine research. In addition, validated clinical outcomes were prioritized, and the certainty of evidence was systematically evaluated using the GRADE approach. The integration of clinical evidence synthesis with exploratory network pharmacology also provides a broader perspective on potential mechanisms.

However, several limitations should be considered. First, all 12 included studies (100%) were conducted in China. This complete geographic concentration substantially limits external validity, as the findings were generated within a single healthcare system and cultural context of traditional medicine [56]. Because LWDH is a traditional East Asian herbal formula, relevant clinical studies are likely to be disproportionately indexed in East Asian databases, which may further contribute to regional publication bias and limit the applicability of findings to non-East Asian populations. Therefore, the pooled estimates may not be directly generalizable to non-Chinese or non-East Asian clinical settings. Second, many studies lacked adequate reporting of key methodological features such as allocation concealment and blinding, resulting in a high or unclear risk of bias. Third, substantial clinical heterogeneity was observed across disease categories, comparator treatments, and intervention protocols. Although further exploration of heterogeneity by disease subtype, baseline severity, or treatment duration would be valuable, the limited number of studies available for MMSE precluded reliable meta-regression. Therefore, the subgroup findings presented in this review should be regarded as exploratory only. Fourth, publication bias could not be robustly assessed for most outcomes because the number of included studies per outcome was insufficient for reliable statistical evaluation. Finally, the network pharmacology analysis was based entirely on computational in silico predictions and was not supported by direct experimental or clinical validation. Therefore, its translational relevance remains limited, and the identified targets and pathways should be interpreted as exploratory hypotheses rather than validated mechanisms.

Future research should focus on conducting well-designed, adequately powered randomized controlled trials with standardized diagnostic criteria and validated outcome measures. Particular attention should be given to disease-specific populations, comparator-specific effects, and long-term outcomes. In addition, experimental studies are required to validate the predicted targets and pathways identified in the network pharmacology analysis and to clarify their biological relevance.

## 4. Materials and Methods

### 4.1. Study Registration

The protocols for the data and methods used in our study were registered in advance with the Open Science Framework and PROSPERO (ID: CRD42024484032).

### 4.2. Data Sources and Search Strategy

The search was conducted on 15 February 2023, and updated on 25 November 2024, to include the most recent information and relevant evidence using a total of 11 databases, including four English databases: MEDLINE (PubMed), EMBASE (Elsevier), the Cochrane Central Register of Controlled Trials (CENTRAL), and CINAHL (Cumulative Index to Nursing and Allied Health Literature) via EMSCO; one Chinese database: the Chinese National Knowledge Infrastructure Database (CNKI); one Japanese database: Citation Information by NII (CiNii); and two domestic databases: Oriental Medicine Advanced Searching Integrated System (OASIS) and Korea Citation Index (KCI). There were no restrictions on the language, publication date, or publication status. The full reproducible search strategies for all databases are provided in Supplementary Content S1 (Supplement File S1).

Because LWDH is a traditional East Asian herbal formula, potentially relevant studies were expected to be disproportionately indexed in Chinese, Korean, and Japanese databases. Therefore, East Asian databases were included to maximize retrieval sensitivity. Notably, despite searching 7 non-East Asian/international databases in addition to 4 East Asian databases, all eligible studies were still derived from China, indicating that the current evidence base is geographically highly concentrated.

### 4.3. Inclusion Criteria

#### 4.3.1. Types of Studies

The selection criteria for the study involved human subjects and Randomized Controlled Trials (RCTs) using random allocation methods. Trials explicitly described as randomized were included to avoid missing potentially relevant studies in a literature base with variable reporting quality. However, when the randomization method was not clearly described, this was conservatively reflected in the risk-of-bias assessment. RCTs that used non-random methods for treatment allocation, such as alternate allocation or allocation based on birth date, were excluded.

#### 4.3.2. Participant Characteristics

The research included studies on patients diagnosed with cognitive impairment using standardized diagnostic tools, regardless of sex, age, or race.

#### 4.3.3. Intervention Types

This review included studies in which LWDH was used as the treatment intervention (Table 3). It encompassed studies that compared HM combined with Western medicine (WM) to WM alone and studies that compared HM combined with WM to HM placebo combined with WM. When the composition of HM was modified to enhance effectiveness, it was explicitly noted as “modified LWDH” (mLWDH). Studies that included the original prescription components of LWDH in their entirety as a treatment intervention were also considered. Regarding the dosage form, only the oral administration of LWDH was considered. Studies that combined LWDH with other treatment methods as interventions were included when other treatment methods were used equally in both the treatment and control groups. Studies that included a placebo or conventional medical treatment as a control group intervention were also included. Studies in which traditional herbal prescriptions were used as control group interventions were excluded as they would not allow for the assessment of the specific effects of LWDH. There were no other restrictions regarding control interventions.

#### 4.3.4. Outcome Measures

##### Cognitive Assessment

The MMSE is a tool used to evaluate cognitive function and assess various areas, including time and place orientation, memory (both short-term and long-term), attention, calculation ability, language, and drawing. Cognitive impairment is distinguished by a score ≤ 23 and normal cognition by a score ≥ 24 (total score range 0–30; higher scores indicate better cognitive function) [58]. The Montreal Cognitive Assessment (MoCA) is an evaluation tool developed to diagnose mild cognitive impairment. It classifies individuals as normal with a score ≥ 23, and as having mild cognitive impairment with a score ≤ 22 (total score range 0–30; higher scores indicate better cognitive abilities) [59]. The ACE-R is a test for measuring cognitive performance and evaluating attention, orientation, memory, fluency, language, and visuospatial abilities. It classifies individuals as normal with a score ≥ 88, possible mild cognitive impairment with a score of 82–87, and abnormal with a score ≤ 81 (total score range 0–100; higher scores indicate better cognitive abilities) [60]. The Alzheimer’s Disease Assessment Scale (ADAS) was developed to assess the severity of cognitive function (ADAS-cognitive subscale or ADAS-Cog) and non-cognitive function (ADAS-noncognitive subscale) in patients with Alzheimer’s disease (total score range 0–85, with higher scores indicating more severe cognitive impairment) [61].

##### Activities of Daily Living (ADLs)

ADLs are an indicator of an individual’s ability to maintain daily self-care. ADLs consist of 14 items. The total score, which was the sum of the scores from the 14 items, was defined as the ‘Activities of Daily Living Functional Index.’ ADLs were assessed using a composite index based on 14 items, with higher scores indicating greater functional impairment [62]. The modified Barthel Index (MBI) is a commonly used scale for measuring disability or dependency in ADLs in stroke survivors (total score range 0–100, with higher scores indicating better ADLs) [63]. The Quality of Life in AD scale evaluates various life domains including physical health, mood, relationships, activities, and ability to complete tasks. It uses a scale of 1–4 (poor, fair, good, or excellent) (total score range 13–52; higher scores indicate a better quality of life) [64].

##### Mental Status

BEHAVE-AD is a tool used to evaluate symptoms in patients with Alzheimer’s disease. It focuses on symptoms that can be treated with medications, categorizing symptoms in Part 1 and providing an overall assessment in Part 2 (total score range: Part 1: 0–75, Part 2: 0–75; higher scores indicate more severe symptoms) [65].

##### Treatment Response

Treatment response was measured using the total effective rate (TER), a composite outcome frequently used in traditional Chinese medicine clinical trials [66]. TER is typically calculated as the proportion of patients classified as “cured,” “significantly improved,” or “improved” among the total sample size [67]. However, because the definition and evaluation criteria of TER vary across studies, and standardized outcome measures are lacking, the interpretation of TER should be approached with caution [68].

##### Traditional Chinese Medicine Symptom Score (TCM)

According to the China Dementia Diagnosis and Treatment Guidelines, the diagnosis of dementia in Traditional Chinese Medicine is identified by symptoms of “insufficiency of the marrow sea (Sui Hai Bu Zu)” and “kidney deficiency (Shen Xu Bu Zu)” [67].

##### Adverse Events

Adverse events observed included dizziness, headache, somnolence, insomnia, nausea, extrapyramidal reactions, and gastrointestinal disturbances.

### 4.4. Data Extraction

Two researchers (HJH and JLP) independently performed data extraction using a standardized data collection form and cross-verified the findings. Discrepancies were resolved through discussion with a third researcher (JTL). The extracted items included the first author’s name, publication year, country, sample size, number of dropouts, detailed information about the participants, HM, control interventions, comparisons, intervention duration, outcome measures, and adverse events related to the intervention. If the data were insufficient or unclear, the corresponding authors of the included studies were contacted via email to request additional information.

### 4.5. Quality Assessment

In this study, the risk of bias was assessed using version 2 of the Cochrane risk-of-bias tool for randomized trials (RoB 2.0) [69] and included in the meta-analysis. Studies were classified as “low risk,” “some concerns,” or “high risk” of bias in each of the following domains: randomization process, deviations from intended intervention, missing outcome data, measurement of the outcome, and selection of the reported result. Two independent researchers (HJH and JLP) evaluated each criterion, and in cases of disagreement, a consensus was reached through discussion between the two researchers and consultation with a third researcher (JTL).

### 4.6. Certainty of Evidence

The quality of evidence for each outcome was assessed using the GRADE [70] through GRADEpro (https://gradepro.org/). The assessment considered the risk of bias, inconsistency, indirectness, and imprecision of results, as well as the potential for publication bias, categorizing the results into one of four levels: “very low,” “low,” “moderate,” or “high.” One researcher (HJH) performed the quality assessment, and another (JLP) cross-verified the data. In case of disagreement, a consensus was reached through discussion between the two researchers and consultation with a third researcher (JTL).

### 4.7. Data Synthesis and Analysis

RStudio (version 2023.06.1+524; Posit, Boston, MA, USA) and R (version 4.3.0; CRAN, WU, Vienna, Austria) were used for data synthesis. The main method for the meta-analysis in the quantitative synthesis utilized a random-effects model, and the results from a fixed-effects model were also presented for sensitivity analysis. The reason for using the random-effects model as the primary model was the potential heterogeneity of treatment regimens and study designs among the included studies in clinical trials of traditional HM, arising from differences in treatment components, study groups, and patient selection criteria. For dichotomous data, relative risk (RR) was used as the primary analysis method, and the odds ratio (OR) was presented as a secondary measure for use in subsequent studies. Point estimates and 95% confidence intervals (CI) were used for reporting. For continuous data, the mean difference (MD) is presented as a 95% CI. Statistical heterogeneity was tested using Higgins I^2^ statistic, with I^2^ ≥ 75% considered indicative of substantial heterogeneity. Subgroup analyses, including (1) duration of treatment, (2) type of WM in the control group, and (3) form of HM, were conducted where necessary data were available, to explain heterogeneity or assess differences between subgroups.

A leave-one-out meta-analysis was conducted to assess the influence of individual studies on the pooled effect estimates and between-study heterogeneity. This involved repeating the meta-analysis while sequentially excluding each study. Through this process, we examined whether the exclusion of any single study materially changed the overall results or contributed to observed heterogeneity. In addition, we evaluated the stability of the pooled estimates when individual studies were removed.

### 4.8. Publication Bias

Funnel plots were used to assess potential publication bias. Asymmetry in the funnel plots was visually examined, and Egger’s regression test was performed to statistically assess funnel plot asymmetry (*p* < 0.05 was considered indicative of potential bias).

The trim-and-fill method was applied as an exploratory approach to estimate the potential impact of publication bias, and funnel plots were generated before and after adjustment.

### 4.9. Gene Set Construction and Intersection Analysis

The chemical components of *Rehmannia glutinosa*, *Dioscorea batatas*, *Cornus officinalis*, *Paeonia suffruticosa*, *Alisma orientale*, and *Poria cocos*, which constitute LWDH, were retrieved from the TM-MC database (https://tm-mc.kr/). Compound–target gene interactions were subsequently identified using the Comparative Toxicogenomics Database (CTD, https://ctdbase.org/).

Disease-associated gene sets for Alzheimer’s disease and vascular dementia were collected from three databases: GeneCards (https://www.genecards.org/), MalaCards (https://www.malacards.org/), and CTD. Given the substantial imbalance in the number of genes and available evidence between Alzheimer’s disease and vascular dementia, different integration strategies were applied.

For Alzheimer’s disease, only genes overlapping across at least two of the three databases were included to reduce potential noise and false-positive associations derived from large-scale text-mining datasets. In contrast, for vascular dementia, all retrieved genes from the three databases were included to avoid underrepresentation due to the relatively limited number of available targets.

The gene sets for Alzheimer’s disease and vascular dementia were then merged and deduplicated to construct a comprehensive cognitive disorder gene set. Intersections between LWDH-associated targets and disease-related gene sets were identified and visualized using a Venn diagram.

To further improve interpretability and reduce noise from large-scale target prediction, the overlapping gene set (n = 7386) was filtered using disease-relevance scores derived from the source databases. This process resulted in a refined set of 1500 genes, which was used for subsequent analyses.

### 4.10. Protein–Protein Interaction (PPI) Network Construction and Modular Clustering

The refined set of 1500 genes associated with LWDH and cognitive disorders was imported into the Metascape platform (v3.5.20240101; https://metascape.org/; accessed 23 February 2026). After mapping to Homo sapiens, 1498 genes were retained for further analysis.

Functional enrichment analysis was performed using multiple databases, including Gene Ontology (GO), Kyoto Encyclopedia of Genes and Genomes (KEGG), Reactome, and WikiPathways. Enriched terms were identified based on statistical significance.

The PPI network was constructed and subsequently analyzed using modular clustering to identify groups of functionally related genes. In this network, nodes represent enriched biological terms, with node size proportional to the number of associated genes and color indicating cluster identity.

The final network structure was visualized using a force-directed layout implemented in Cytoscape (v3.10.4; Cytoscape Consortium, San Diego, CA, USA) through the Metascape platform.

### 4.11. Functional Annotation and Tissue-Specific Enrichment Analysis

Based on the clustering results, Cluster 8, which showed enrichment for pathways related to Alzheimer’s disease, was selected for further analysis. A total of 226 genes within this cluster were analyzed using ShinyGO v0.82 (https://bioinformatics.sdstate.edu/go/) for Gene Ontology (GO) and KEGG pathway enrichment analysis. The top 20 enriched pathways were visualized using dot plots.

To assess tissue-specific expression patterns, the same gene set was further analyzed using the Human Gene Atlas and GTEx datasets available through the Enrichr platform (https://maayanlab.cloud/Enrichr/; accessed 23 February 2026). This analysis was conducted to explore whether the identified genes show preferential expression in brain tissues relevant to cognitive function.

## 5. Conclusions

LWDH may offer potential adjunctive benefits in improving cognitive function and daily functioning in patients with cognitive impairment associated with neurodegenerative disorders. However, the current evidence is limited by methodological weaknesses, risk of bias, and substantial clinical heterogeneity.

Therefore, the findings of this study should be interpreted cautiously, and definitive conclusions regarding clinical effectiveness cannot be drawn. The network pharmacology results provide exploratory biological context but do not establish causal mechanisms.

Further high-quality, disease-specific, and comparator-specific clinical trials are required to confirm the potential role of LWDH as an adjunctive treatment for cognitive impairment.

## Figures and Tables

**Table 1:** Characteristics of the included studies.

Study ID	Sample Size (A):(B) (Included → Analyzed)	Sex (Male/ Female)	Age (Mean ± SD or Range) (yr)	Disease Duration (Mean ± SD) (yr)	(A) Treatment Group	(B) Control Group	Treatment/Follow-Up Period	Outcome and Results	Adverse Events (A):(B) (%)
Shen et al. 2013 [36]	110 (55:55) → 110 (55:55)	(A) 32/23 (B) 34/21	(A) 62–83 (75.3) (B) 61–83 (74.6)	(A) 1–7 (3.8) AD 27 VD 28 (B) 2–7 (4.1) AD 29 VD 26	LWDH pill 10 g bid + (B)	Olanzapine tablets ^†^ 2.5 mg qd Adjusted based on symptoms (5–20 mg)	3 m/NA	(1) TER (Behave-AD) * (2) MMSE (3) BEHAVE-AD (4) Side effect *	(a) 1:5 (b) 2:4 (c) 2:3 (d) 2:5
Duan et al. 2014 [37]	120 (60:60) → 120 (60:60)	76/44	61–88 (75.56)	2–7 (3.8 ± 2.7)	LWDH pill 10 g bid + (B)	Olanzapine tablets ^†^ 2.5 mg qd Adjusted according to symptoms (5–20 mg)	3 m/NA	(1) TER (Behave-AD) (2) MMSE (3) BEHAVE-AD (4) Side effect *	NR
Du et al. 2015 [38]	105 (51:54) → 105 (51:54)	(A) (31/20) (B) (32/22)	(A) 63–83 (74) (B) 60–83 (74.7)	(A) 1–6 (3.7) AD 28 VD 23 (B) 1–6.5 (3.9) AD 29 VD 25	LWDH 3 T bid + (B)	Memantine 1 w 0.5 T 5 mg OMP 2 w 10 mg 0.5 T bid 3 w 15 mg (10/5 bid) 4 w 10 mg bid	3 m/NA	(1) TER (Behave-AD) * (2) BEHAVE-AD (3) ADL * (4) ACE-R (5) Side effects *	(a) 1:4 (b) 2:4 (c) 1:3 (d) 3:5
Yu et al. 2017 [39]	120 (60:60) → 120 (60:60)	(A) (35/25) (B) (34/26)	(A) 72.5 ± 2.7 (B) 72.4 ± 2.8	AD (A) 1–6 (3.8) (B) 1–6 (3.9)	LWDH # 100 mL bid + (B)	Donepezil Hydrochloride Tablets 5 mg qd	NR/NR	(1) TER (Behave-AD) * (2) BEHAVE-AD (3) ADL * (4) ACE-R (5) side effect *	(a) 1:4 (b) 2:4 (c) 1:3 (d) 2:5
Chen et al. 2017 [40]	70 (35:35) → 70 (35:35)	(A) (21/14) (B) (23/12)	(A) 58–81 (72.3) (B) 61–83 (73.6)	(A) 0.5–5.5 (3.5) 18 AD 17 VD (B) 1.0–6.0 (3.7) 25 AD 10 VD	LWDH 6 g bid + (B)	Donepezil Hydrochloride Tablets 5 mg qd 8 w 10 mg qd 16 w	24 w/NA	(1) TER (Behave-AD) * (2) BEHAVE-AD (3) ADL * (4) ACE-R	No adverse reactions
Zhang et al. 2017 [41]	80 (40:40) → 80 (40:40)	(A) (26/14) (B) (23/17)	(A) 42–79 (65 ± 4.1) (B) 46–82 (67.1 ± 5.3)	NR	mL WDH # bid + (B)	Donepezil Hydrochloride Tablets 5 mg qd	8 w/NA	(1) TER (MMSE) * (2) MMSE * (3) ADL *	NR
Zhang et al. 2017 [42]	76 (38:38) → 76 (38:38)	(A) (19/19) (B) (17/21)	(A) 70.3 ± 5.8 (B) 69.4 ± 6.3	NR	LWDH 8 T tid + (B)	Donepezil Hydrochloride Tablets 5 mg OS1 4 w 10 mg OS1 16 w	20 w/NA	(1) TER * (2) MMSE * (3) ADL * (4) ADAS-cog * (5) TCMs *	NR
Suns et al. 2020 [29]	110 (55:55) → 110 (55:55)	(A) (29/26) (B) (30/25)	(A) 72.6 ± 5.5 (B) 61–85 (73.0 ± 5.2)	(A) 0.5~5.4 (29 ± 0.0) (B) 0.4–5.2 (2.8 ± 0.2) VD 24 AD 20 Mixed 11	LWDH pill 3 T bid + (B)	Memantine 1 w 0.5 T 5 mg qd 2 w 10 mg 0.5 T bid 3 w 15 mg (10/5 bid) 4 w 10 mg bid6	3 m/NA	(1) MMSE * (2) ADL * (3) ADAS-cog * (4) QOL-AD * (5) oxidative stress index * (MDA, SOD, GSH-Px) (6) Side Effect *	(a) 2:7 (b) 3:1 (c) 1:4 (d) 1:2
Jin et al. 2020 [43]	80 (40:40) → 80 (40:40)	(A) (26/14) (B) (24/16)	(A) 62–83 (73.2 ± 1.5) (B) 61–82 (72.8 ± 1.5)	NR	LWDH pill 6 g bid + (B)	Donepezil Hydrochloride Tablets 5 mg OS1 8 w 10 mg OS1 8~16 w	16 w/NA	TER (Behave-AD) *	NR
Song et al. 2021 [31]	106 (53:53) → 106 (53:53)	(A) (24/29) (B) (22/31)	(A) 52–86 (68.83 ± 5.95) (B) 69.51 ± 6.06	AD (A) 1.5–10.8 (6.14 ± 1.81) (B) 1.0–12.5 (6.73 ± 2.01)	LWDH pill 8 T tid + (B)	Hypertension, Hyperliqidemia, nerve nutrition Memantine 1 w 5 mg qd 2 w 5 mg bid 3 w (10 mg/5 mg) bid 4 w 10 mg bid	6 m	(1) TER (Behave-AD) * (2) MMSE * (3) Behave-AD * (4) ADL * (5) QOL-AD * (6) BDNF, Aβ, IL-1β (7) Side Effect	(a) 2:1 (b) 1:0 nausea and vomiting 2:2 gastrointestinal reaction 1:1
Li 2022 [30]	110 (56:54) → 100 (51:49)	(A) (31/25) (B) (30/24)	(A) 65–80 (70.29 ± 6.71) (B) 70.62 ± 6.58	18 < MoCA < 26 21 < MMSE < 26 (A) HTN 9–28 y (14.15 ± 2.43 y) (B) HTN 7–16 y (13.94 ± 2.31 y)	mLWDH Granules 8 mg tid + antihypertensive drugs (ARB, BB, CCB)	Placebo LWDH Granules 8 mg tid + antihypertensive drugs (ARB, BB, CCB)	4 m/6 m	(1) TER(MoCA) * (2) MMSE * (3) MoCA (4) ADL (IADL, BADL) * (5) BP (24 h SSD, 24 h DSD, nSCV, nDCV) * (6) TCMs (Kidney Yin Deficiency Syndrome score) * (7) serum protein markers (Aβ1–42, total Tau protein, P-tau) * (8) vascular inflammatory markers (Lp-PLA2, PTX3, Hcy) * (9) circulating miR-126 and miR-155 *	No adverse reactions
Rao et al. 2024 [44]	60 (30:30) → 60 (30:30)	(A) (15/15) (B) (14/16)	(A) 60–85 (69.3 ± 5.9) (B) 60–85 (68.4 ± 6.5)	AD (A): ND (B): ND	LWDH pill 8 T tid + (B)	Donepezil Hydrochloride Tablets 5 mg qd After one month adjust according to symptoms (5–10 mg)	3 m/NA	(1) TER(TCM) (2) TCM (3) MMSE (4) ADL	NR

A, treatment group; B, control group; NA, not available; NR, not reported. Definition of each side effect: (a) dizziness and headache, (b) drowsiness, (c) insomnia, and (d) extrapyramidal adverse events; symptoms such as dystonia, gait disturbance, and inability to remain seated are also referred to as Parkinsonian symptoms. *, statistically significant (*p* < 0.05). # Detailed personalized prescriptions have not been described. ^†^: One of the antipsychotic drugs used for BPSD. Prevention and Treatment of Ischemic Neurological Disorders Due to Cerebral Vasospasm After Aneurysmal Subarachnoid Hemorrhage. Abbreviations: BB, beta-blocker; ARB, angiotensin receptor blocker; CCB, calcium channel blocker; Hcy, homocysteine; MDA, malondialdehyde; BDNF, brain-derived neurotrophic factor; miR, microRNA; P-tau, phosphorylated tau; PTX3, pentraxin 3; SOD, superoxide dismutase; IL-1β, interleukin-1 beta; Lp-PLA2, lipoprotein-associated phospholipase A2; GSH-Px, glutathione peroxidase; Aβ, amyloid beta.

**Table 2:** Quality assessment of evidence (GRADE).

Certainty Assessment							№ of Patients		Effect		Certainty	Importance
№ of Studies	Study Design	Risk of Bias	Inconsistency	Indirectness	Imprecision	Other Considerations	LWDH + WM	WM	Relative (95% CI)	Absolute (95% CI)		
cognition (follow-up: range 8 weeks to 6 months; assessed with: MMSE)												
7	randomized trials	serious ^a^	serious ^b^	not serious	serious ^c^	none	331	331	-	MD 2.34 higher (0.88 higher to 3.79 higher)	⨁◯◯◯ Very low ^a,b,c^	CRITICAL
ADL (follow-up: range 8 weeks to 6 months; assessed with: MBI)												
5	randomized trials	serious ^a^	not serious	not serious	serious ^c^	none	234	237	-	MD 10 SD higher (9.12 higher to 10.88 higher)	⨁⨁◯◯ Low ^a,c^	CRITICAL
Treatment effective rate (follow-up: range 4 weeks to 12 weeks; assessed with: symptoms)												
10	randomized trials	serious ^a^	not serious	not serious	not serious	none	382/432 (88.4%)	377/495 (76.2%)	RR 1.12 (1.04–1.22)	91 more per 1000 (from 30 more to 168 more)	⨁⨁⨁◯ Moderate ^a^	CRITICAL
Symptom Category Scores of the Behavior Pathology in Alzheimer’s Disease (follow-up: range 3–6 months; assessed with: BEHAVE-AD)												
6	randomized trials	serious ^a^	serious ^b^	not serious	serious ^c^	none	314	317	-	MD 0.58 lower (1.8 lower to 0.64 higher)	⨁◯◯◯ Very low ^a,b,c^	CRITICAL
Adverse effects (follow-up: range 8 weeks to 6 months)												
6	randomized trials	serious ^a^	not serious	not serious	serious ^c^	none	26/309 (8.4%)	66/312 (21.2%)	RR 0.40 (0.26–0.61)	127 fewer per 1000 (from 157 fewer to 83 fewer)	⨁⨁◯◯ Low ^a,c^	CRITICAL

Explanations: ^a^. Blinding was not reported in the included studies. ^b^. If the heterogeneity of the estimated value was >75%, it was lowered by one level. ^c^. Dichotomous variables were lowered by one level when there were <300 people per group, and continuous variables were lowered by one level when there were <400 people per group. ⨁, level of certainty of evidence; ◯, downgraded level of certainty.

**Table 3:** Botanical drugs in Liuwei Dihuang (LWDH) and their doses for single administration.

Scientific Name with [Botanical Drug Name]	Pinyin Name	Parts Used	Single Dose (g)
			Granule
Liuwei Dihuang (LWDH)			
*Rehmannia glutinosa* (Gaertn.) DC. [Orobanchaceae; Rehmanniae Radix Preparata]	Shu Di Huang	Steamed and dried Rhizome	30
*Dioscorea polystachya* Turcz. [Dioscoreaceae; Dioscoreae Rhizoma]	Shan Yao	Periderm-removed rhizome (subterranean stem)	15
*Cornus officinalis* Sieblod and Zucc. [Cornaceae; Corni Fructus]	Shan Zhu Yu	Dried fruit with the seeds removed	15
*Paeonia* × *suffruticosa* Andrews [Paeoniaceae; Moutan Radicis Cortex]	Mu Dan Pi	Dried Root Bark	11.25
*Poria cocos* (Schw.) Wolf [Polyporaceae; Poria Sclerotium]	Fu Ling	Dried sclerotium	11.25
*Alisma plantago-aquatica* subsp. *orientale* (Sam.) Sam. [Alismataceae; Alismatis Rhizoma]	Ze Xie	Dried tuber	11.25

The composition and dosage were summarized based on previous literature [57]. Traditional units (liang) were converted based on commonly used modern standards (1 liang ≈ 37.5 g).

## Data Availability

No new data were created or analyzed in this study. Data sharing is not applicable.

## Supplementary Materials

The following supporting information can be downloaded at https://www.mdpi.com/article/10.3390/ph19050776/s1: Supplementary Content S1: Search strategies; Supplementary Content S2: Details of excluded reports; Supplementary Content S3: Diagnostic references of included studies; Supplementary Content S4: Herbal composition and dosages; Supplementary Content S5: Total effective rate criteria; Supplementary Content S6: Additional forest plots; Supplementary Content S7: Leave-one-out sensitivity analyses. Table S1: Comparison with the Herb and Disease gene; Table S2: 1500 Core target genes with the disease relevance score; Table S3: Network cluster analysis and functional enrichment analysis in Metascape; Table S4: 266 core genes from Cluster 8; Table S5: Enrichment analysis of Cluster 8-cored genes in Human Gene Atlas database by Enrichr. References [71,72,73,74,75,76,77,78,79,80,81,82,83,84,85,86,87,88,89,90,91,92,93,94,95,96,97,98] cited in Supplementary Materials.

## Abbreviations

The following abbreviations are used in this manuscript:

AD	Alzheimer’s Disease
ADAS-cog	cognitive portion of the Alzheimer’s Disease Assessment Scale
ADL	Activities of Daily Living
Aβ	Amyloid beta
BADL	Basic Activity of Daily Living
Behave-AD	The Behavioral Pathology in Alzheimer’s Disease Rating Scale
BDNF	Brain-derived neurotrophic factor
BPRS	Brief Psychiatric Rating Scale
CCMD	Classification and Diagnosis of Mental Disorders in China Diagnosis
DSM-IV	Diagnostic and Statistical Manual of Mental Disorders [of APA] fourth edition
IADL	instrumental activities of daily living
mLWDH	modified Liuwei Dihuang
MMSE-R	MMSE results
MMSE	Mini-Mental State Examination
MoCA	Montreal Cognitive Assessment
NIA-AA	National Institute of Ageing-Alzheimer Association
QOL-AD	Quality of Life in Alzheimer’s Disease
TCM	Traditional Chinese Medicine
TCMs	Traditional Chinese Medicine symptoms
TER	Total Effective Rate
TIR	Total incidence rate
VD	vascular dementia
WM	Western medicine
