# Supplementary material for: Effectiveness and Safety of Liuwei Dihuang as an Adjunctive Therapy for Cognitive Impairment: A Systematic Review, Meta-Analysis, and Network Pharmacology Analysis
Source: Pharmaceuticals (Basel). 2026 May 15;19(5):776. doi: 10.3390/ph19050776 (PMC13209968; doi:10.3390/ph19050776)
Supplement: Supplementary file 1 [file pharmaceuticals-19-00776-s001.zip › File S1-LWDH_2025.pdf]

Supplementary Material for Effectiveness and Safety of Liuwei Dihuang Decoction/Pill on Cognitive Disorder: A Systematic Review and Meta-Analysis.

Supplementary Content S1. Search strategies used in each database

MEDLINE via PubMed

	Searches	Results
**#1**	Dementia[MeSH] OR dement*[Title/Abstract] OR Alzheimer*[Title/Abstract] OR "Lewy body"[Title/Abstract] OR Huntington*[Title/Abstract] OR Parkinson*[Title/Abstract] OR "Pick disease"[Title/Abstract] OR "cognitive impairment"[Title/Abstract] OR "vascular dementia"[Title/Abstract] OR "Post-stroke dementia"[Title/Abstract] OR "Post-stroke cognitive impairment"[Title/Abstract]	528,786
**#2**	liuwei dihuang pill[TIAB] OR Liuwei Dihuang Wan[TIAB] OR Liuwei Dihuang Bolus[TIAB] OR decoction of six drugs containing rhigoma rehmanine[TIAB] OR Liuwei Dihuang decoction[TIAB] OR LiuWei DiHuang Tang[TIAB] OR Yookmijihwangtang[TIAB] OR Liuwei Dihuang tang[TIAB] OR Yukmijihwang-tang[TIAB] OR Yukmijihwangtang-gamibang[TIAB] OR Yugmijihwangtang[TIAB] OR Yukmigeehwang-hwan[TIAB] OR Yukmijihwanghuan[TIAB] OR Yukmijihwang-Hwan[TIAB] OR Yukmiziwhang-Whan[TIAB] OR Yukmichiwhang-won[TIAB] OR Rokumigan[TIAB] OR TJ-87[TIAB] OR Rokumi*[TIAB]	173
**#3**	#1 AND #2	21

EMBASE via Elsevier

	Searches	Results
**#1**	'dementia'/exp OR 'dement*':ab,ti OR 'Alzheimer disease'/exp OR 'Alzheimer*':ab,ti OR 'Lewy body'/exp OR 'Lewy body':ab,ti OR 'Huntington*':ab,ti OR 'Parkinson disease'/exp OR 'Parkinson':ab,ti OR 'Pick disease':ab,ti OR 'cognitive impairment':ab,ti OR 'vascular dementia':ab,ti OR 'Post-stroke dementia':ab,ti OR 'post-stroke cognitive impairment':ab,ti	725,413

**#2**	"iuwei dihuang pill" OR "Luwei Dihuang Wan" OR "Liuwei Dihuang Bolus" OR " decoction of six drugs containing rhigoma rehmanine" OR "Liuwei Dihuang decoction" OR "LiuWei DiHuang Tang" OR "Yookmijihwangtang" OR "Liuwei Dihuang tang" OR "Yukmijihwang-tang" OR "Yukmijihwangtang-gamibang" OR "Yugmijihwangtang" OR "Yukmigeehwang-hwan" OR "Yukmijihwanghuan" OR "Yukmijihwang-Hwan" OR "Yukmiziwhang-Whan" OR "Yukmichiwhang-won" OR "TJ-87" OR "Rokumi"	146
**#3**	#1 AND #2	24

CENTRAL

	**Searches**	**Results**
**#1**	MeSH descriptor: [Dementia] explode all trees	28830
**#2**	(dement* OR Alzheimer* OR "Lewy body" OR Huntington* OR Parkinson* OR "Pick disease" OR "cognitive impairment" OR "vascular dementia" OR "Post-stroke dementia" OR "post-stroke cognitive impairment"):ti,ab,kw	41866
**#3**	(Liuwei dihuang pill OR Luwei Dihuang Wan OR Liuwei Dihuang Bolus OR decoction of six drugs containing rhigoma rehmanine OR Liuwei Dihuang decoction OR LiuWei DiHuang Tang OR Yookmijihwangtang OR Liuwei Dihuang tang OR Yukmijihwang-tang OR Yukmijihwangtang-gamibang OR Yugmijihwangtang OR Yukmigeehwang-hwan OR Yukmijihwanghuan OR Yukmijihwang-Hwan OR Yukmiziwhang-Whan OR Yukmichiwhang-won OR Rokumigan OR TJ-87 OR Rokumi)ti,ab,kw	0
**#4**	(#1 OR #2)AND(#3)in Trials	0

CINAHL via EBSCO

	**Searches**	**Results**

**#1**	Dementia OR dement* OR Alzheimer* OR “Lewy body” OR Huntington* OR Parkinson* OR “Pick disease” OR “cognitive impairment” OR “vascular dementia” OR “Post-stroke dementia” OR “post-stroke cognitive impairment”	174,627
**#2**	liuwei dihuang pill[TX] OR Liuwei Dihuang Wan[TX] OR Liuwei Dihuang Bolus[TX] OR decoction of six drugs containing rhigoma rehmanine[TX] OR Liuwei Dihuang decoction[TX] OR LiuWei DiHuang Tang[TX] OR Yookmijihwangtang[TX] OR Liuwei Dihuang tang[TX] OR Yukmijihwang-tang[TX] OR Yukmijihwangtang-gamibang[TX] OR Yugmijihwangtang[TX] OR Yukmigeehwang-hwan[TX] OR Yukmijihwanghuan[TX] OR Yukmijihwang-Hwan[TX] OR Yukmiziwhang-Whan[TX] OR Yukmichiwhang-won[TX] OR Rokumigan[TX] OR TJ-87[TX] OR Rokumi[TX]	68
**#3**	#1 AND #2	9

OASIS KCI

	Searches	Results
**#1**	(치매 OR 인지장애 OR 혈관성치매 OR 알츠하이머) AND 육미	0

Note: The non-English search terms were retained exactly as used in the original database searches to ensure reproducibility. English translations are provided as follows:

치매 = dementia; 인지장애 = cognitive impairment; 혈관성치매 = vascular dementia; 알츠하이머 = Alzheimer's disease; 육미 = Liuwei.

CNKI

	Searches	Results
**#1**	(SU='痴呆'+'阿尔茨海默病'+’阿尔兹海默症’+'dementia'+'Alzheimer'+’血管性痴呆’+’vascular dementia’+'认知障碍'+’ 老年痴呆’+’老年性痴呆’+’混合型痴呆’) AND (SU=’ '六味地黄方'+'六味地黄丸'+'六味地黄湯'+'六味地黄'+’六味地黄汤'+'六味地黄散'+'六味地黄颗粒'+'六味地黄胶囊’+’六味丸’+’Rokumigan’+’六味’+’Rokumi’+’TJ-87’)	1,137

Note: The Chinese search terms were retained exactly as used in the original database search to ensure reproducibility. English translations are as follows: 痴呆 (dementia); 阿尔茨海默病 and 阿尔兹海默症 (Alzheimer's disease); 血管性痴呆

(vascular dementia); 认知障碍 (cognitive impairment); 老年痴呆 and 老年性痴呆 (senile dementia); 混合型痴呆 (mixed dementia); 六味地黄方 (Liuwei Dihuang formula); 六味地黄丸 (Liuwei Dihuang Pill); 六味地黄湯 and 六味地黄汤 (Liuwei Dihuang Decoction); 六味地黄散 (Liuwei Dihuang Powder); 六味地黄颗粒 (Liuwei Dihuang Granules); 六味地黄胶囊 (Liuwei Dihuang Capsules); 六味丸 (Liuwei Pill); 六味 (Liuwei).

CINII

	**Searches**	**Results**
**#1**	(認知症 OR 脳血管性認知症 OR 年性痴呆症 OR 痴呆症 OR 痴呆 OR 老人性痴呆 OR アルツハイマ) AND (六味地黄方 OR 六味地黄 OR 六味地黄丸 OR 六味地黄湯 OR 六味地黄散 OR 六味地黄颗粒 OR 六味地黄胶囊 OR 六味丸 OR Rokumigan OR 六味 OR Rokumi OR TJ-87)	1

Note: The Japanese and Chinese search terms were retained exactly as used in the original database search to ensure reproducibility. English translations are as follows: 認知症 (dementia); 脳血管性認知症 (vascular dementia); 年性痴呆症, 痴呆症, 痴呆, and 老人性痴呆 (senile dementia); アルツハイマ (Alzheimer's disease); 六味地黄方 (Liuwei Dihuang formula); 六味地黄 (Liuwei Dihuang); 六味地黄丸 (Liuwei Dihuang Pill); 六味地黄湯 (Liuwei Dihuang Decoction); 六味地黄散 (Liuwei Dihuang Powder); 六味地黄颗粒 (Liuwei Dihuang Granules); 六味地黄胶囊 (Liuwei Dihuang Capsules); 六味丸 (Liuwei Pill); 六味 (Liuwei).

Supplementary Content S2. Details of excluded reports

1. Nonrandomized control trial (n = 4) [1–4]

2. Case report (n =9) [5–13]

3. Review article (n = 3) [14–16]

4. not about LWDH (n=5) [17–21]

5. comparison of different CMS (n=4) [22–25]

6. Head to head (n=2) [26,27]

7. not available full-text (n=1) [28]

Supplementary Content S3. Details of diagnostic references of included studies

Study ID	Diagnostic Criteria
**2013_Shen[29]**	(1) CCMD-3 (Classification and Diagnostic Criteria of Mental Disorders in China;《中國精神疾病分類方案與診斷標準》)<br>(2) Dementia psychiatric symptoms<br>(3) Duration of disease ≤ 5 years<br>(4) GDS＜5, Dementia Behavior Scale (BEHAVE-AD) score ≥ 8
**2014_Duan[30]**	(1) VD, AD (ND)
**2015_Du[31]**	(1) DSM-IV《精神障碍诊 断和统计手册》<br>(2) "Standards for Diagnosis, Dialectical Classification and Efficacy Evaluation of Alzheimer's Disease《老年呆病的诊断、辩证分型及疗效评定标准》"<br>"Guiding Principles for Clinical Research of New Chinese Medicines for Dementia Treatment《中药新药 治疗痴呆临床研究指导原则》"<br>(3) BPSD<br>(4) Duration of disease ≤ 5 years<br>(5) GDS < 5, BEHAVE-AD ≥ 8
**2017_Yu[32]**	(1) Alzheimer's disease (NR)

**2017_Chen**[33]	(1) DSM-IV《精神障碍诊 断和统计手册》<br>(2) MMSE<br>(3) MRI,CT<br>(4) Guiding Principles for Clinical Research of New Drugs of Traditional Chinese Medicine (Trial Implementation)《中药新药临床研究指导原则(试行)》
**2017_Zhang_1**[34]	(1) DSM-IV of vascular dementia《精神障碍诊 断和统计手册》<br>(2) CT MRI _ Evidence of cerebrovascular disease<br>(3) Dementia within 3 months of stroke.
**2017_Zhang_2**[35]	(1) NIA-AA<br>(2) CDR<br>(3) China Dementia Treatment Guidelines (sea of marrow deficiency, kidney deficiency)《中国痴呆诊疗指南》
**2020_jin**[36]	(1) DSM-IV《精神障碍诊 断和统计手册》<br>(2) “Guiding Principles for Clinical Research of New Drugs of Traditional Chinese Medicine”_Alzheimer”s disease of kidney yin deficiency《中药新药临床研究指导原则》
**2020_Suns**[37]	(1) CCMD-3《中國精神疾病分類方案與診斷標準》<br>(2) BEHAVE-AD ≤8

**2021_Song[38]**	(1) "Consensus on the Diagnosis and Treatment of Alzheimer's Disease in Traditional Chinese Medicine"《阿尔茨海默病 的中医诊疗共识》<br><br>(2) BEHAVE-AD ≥ 8 points<br><br>(3) MMSE < 20
**2022_LI[39]**	(1) "Guidelines for the Management of Hypertension in the Elderly 2019"《中国老年高血压管 理指南 2019》 Age ≥ 65 years SBP ≥ 140 mmHg and/or DBP ≥ 90 mmHg<br><br>(2) "2018 Chinese Guidelines for the Diagnosis and Treatment of Dementia and Cognitive Impairment : Dementia and Its Classification and Diagnostic"《2018 中国痴呆与认知障 碍诊治指南 (一) : 痴呆及其分类诊断标准<br><br>(3) "Diagnostic Guidelines for Common Diseases in Internal Medicine of Traditional Chinese Medicine - Part of Western Medicine Diseases"《中医内科常见病诊断指 南西医疾病部分》<br><br>(4) "Vascular Dementia Diagnosis, Syndrome Differentiation and Curative Effect Judgment Criteria Establishment《血管性痴呆的诊断、辨证及疗效判定标准制定》"
**2024_RAO[40]**	(1) "2018 Chinese Guidelines for the Diagnosis and Treatment of Dementia and Cognitive Impairment : Dementia and Its Classification and Diagnostic" 《2018 中国痴呆与认知障碍诊治指南(二) : 阿尔茨海默病诊治指南》<br><br>(2) "Consensus on the Diagnosis and Treatment of Alzheimer's Disease in Traditional Chinese Medicine"《阿尔茨海默病的中医诊疗共识》<br><br>(3) HIS≤7<br><br>(4) HAMD≤7<br><br>(5) CDR≥1.0

*Footnote

AD: Alzheimer's Disease, BEHAVEAD: The Behavioral Pathology in Alzheimer's Disease Rating Scale, BPRS: Brief Psychiatric Rating Scale, BPSD: Behavioral and psychological symptoms of dementia, MRI: magnetic resonance imaging, CCMD: Classification and Diagnosis of Mental Disorders in China Diagnosis, CDR: clinical dementia rating scale, CT: computed tomography, dbp: diastolic blood pressure, dsm-IV: Diagnostic and Statistical Manual of Mental Disorders [of APA], fourth edition, GDS: Global Deterioration Scale, HIS: health information system, HAMD: Hamilton Depression Rating Scale, MMSE: Mini Mental State Examination, ND: not detailed, NIA-AA: National Institute of Ageing-Alzheimer Association, SBP: systolic blood pressure, VD: vascular dementia.

Supplementary Content S4. Details of herbs usage in included studies

Scientific name	Chinese name (Pinyin name)	2013_Shen	2014_Duan	2015_Du	2017_ Yu#	2017_Chen	2017_Zhang	2017_Zhang#	2020_Jin	2020_Suns	2021_ Song	2022_LI	2024_RAO
Rehmannia glutinosa	熟地黃(Shu Di Huang)	N/R	N/R	N/R	15 g	N/R	N/R	25 g	N/R	N/R	N/R	30 g	N/R
Cornus officinalis	山茱萸（酒制）(Shan Zhu Yu)	N/R	N/R	N/R	12 g	N/R	N/R	15 g	N/R（制）	N/R（制）	N/R（制）	10 g	N/R
Dioscorea batatas	山藥(Shan Yao)	N/R	N/R	N/R	12 g	N/R	N/R	15 g	N/R	N/R	N/R	15 g	N/R
Poria cocos	白茯苓(Fu Ling)	N/R	N/R	N/R	10 g	N/R	N/R	15 g (茯神)	N/R	N/R	N/R	15 g	N/R
Paeonia suffruticosa	牧丹皮(Mu Dan Pi)	N/R	N/R	N/R	10 g	N/R	N/R	12 g	N/R	N/R	N/R	10 g	N/R
Alisma orientalis	澤瀉(Ze Xie)	N/R	N/R	N/R	10 g	N/R	N/R	ND	N/R	N/R	N/R	10 g	N/R

Cuscuta chinensis	菟絲子(Tǔ Sī Zǐ)		20 g
Curcuma longa	姜黃(Jiāng Huáng)		10 g
Salvia miltiorrhiza	丹蔘(Dān Shēn)	12 g	15 g
Polygala tenuifolia	遠志(Yuán Zhì)		10 g
Acorus tatarinowii	石菖蒲(Shí Chāng Pǔ)	15 g	10 g
Rhodiola rosea	紅景天(Hóng Jīng Tiān)		10 g
Plantago asiatica	車前子(Chē Qián Zǐ)		
Cinnamomi Cortex	肉桂(Ròu Guì)		10 g

Alpinia oxyphylla Miq	.益智仁(Yì Zhì Rén)				12 g	
Lycium chinense	枸杞子(Gǒu Qǐ Zǐ)				12 g	
Euphoria longan	龍眼肉(Lóng Yǎn Ròu)				10 g	
Panax ginseng	人蔘(Rén Shēn)				5 g	
Glycyrrhiza uralensis	炙甘草(Zhì Gān Cǎo)				6 g	
mel	蜂蜜(Fēng Mì)	N/R	N/R	N/R		N/R

*footnote

Included studies are listed in References [29–40]

#: Herbal prescriptions modified according to symptoms

2017_ Yu#

风证者加生龙骨、生牡蛎、菊花等；兼痰证者加郁金、陈皮、清半夏等 For wind syndrome (风证), add Sheng Long Gu (Os Draconis), Sheng Mu Li (Ostrea gigas), and Ju Hua (Chrysanthemum morifolium), among others. For concurrent phlegm syndrome (痰证), add Yu Jin (Curcuma longa), Chen Pi (Citrus reticulata), and Qing Ban Xia (Pinellia ternata), among others.

For blood stasis syndrome (瘀血证), add 当归 (*Angelica sinensis*) and 三七 (*Panax notoginseng*) to promote blood circulation and resolve stasis (活血化瘀).
For liver qi stagnation (肝郁), add 柴胡 (*Bupleurum chinense*) and 白芍 (*Paeonia lactiflora*) to soothe the liver and relieve depression (疏肝解郁).

2017_Zhang#

For wind syndrome (风证), add 生龙骨 (*Os draconis*), 生牡蛎 (*Ostrea gigas*), 菊花 (*Chrysanthemum morifolium*). and others.

For concurrent phlegm syndrome (痰证), add 郁金 (*Curcuma longa*), 陈皮 (*Citrus reticulata*), 清半夏 (*Pinellia ternata*), and others.

火证者加黄芩、黄连、竹茹等；兼气虚者加黄芪、焦白术等

For fire syndrome (火证), add 黄芩 (*Scutellaria baicalensis*), 黄连 (*Coptis chinensis*), 竹茹 (*Bambusa vulgaris*), and others.
For concurrent qi deficiency (气虚), add 黄芪 (*Astragalus membranaceus*), 焦白术(*Atractylodes macrocephala* (roasted)), and others.

血瘀者加赤芍、乳香、没药等

For blood stasis syndrome (血瘀), add 赤芍 (*Paeonia veitchii*), 乳香 (*Boswellia serrata*), 没药 (*Commiphora wightii*), and others.

Supplementary Content S5. Details of description of total effective rate of included studies

Study ID	TER (Total effective rate)
2013_Shen	① 痊愈(Cured) BEHAVE-AD score reduction rate ≥75%<br>② 显效(Markedly effective): BEHAVE-AD scoring table reduction rate ≥50%, <75%<br>③ 有效(Effective): BEHAVE-AD score reduction rate ≥25%, <50%<br>④ 无效(invalid): BEHAVE-AD score reduction rate <25%
2014_Duan	① 痊愈(Cured) BEHAVE-AD score reduction rate ≥75%<br>② 显效(Markedly effective): BEHAVE-AD scoring table reduction rate ≥50%, <75%<br>③ 有效(Effective): BEHAVE-AD score reduction rate ≥25%, <50%<br>④ 无效(invalid): BEHAVE-AD score reduction rate <25%
2015_Du	① 痊愈(Cured) BEHAVE-AD score reduction rate ≥75%<br>② 显效(Markedly effective): BEHAVE-AD scoring table reduction rate ≥50%, <75%<br>③ 有效(Effective): BEHAVE-AD score reduction rate ≥25%, <50%<br>④ 无效(invalid): BEHAVE-AD score reduction rate <25%
2017_Yu	① 痊愈(cured) reduction rate of BEHAVE-AD score ≥75%;<br>② 显效(markedly effective): 75%> BEHAVE-AD score reduction rate ≥50%;<br>③ 有效(Valid): 50%> BEHAVE-AD score reduction rate ≥25%;<br>④ 无效(Invalid): reduction rate of BEHAVE-AD score <25%
2017_Chen	① 显效(Markedly effective): BEHAVE-AD score reduction rate ≥50%<br>② 有效(Effective): 50%> BEHAVE-AD score reduction rate ≥25%<br>③ 无效(Ineffective): BEHAVE-AD score reduction rate <25%.<br><br>Total effective rate = (marked effect + effective) / total number of cases.

2017_Zhang_1	MMSE curative effect index = (integral after treatment – integral before treatment)/integral before treatment × 100%.<br><br>① 显效(Markedly effective): MMSE curative effect index ≥20%<br>② 有效(Effective): 12% ≤ MMSE curative effect index <20%<br>③ 无效(Ineffective): 0 ≤ MMSE curative effect index <12%<br>④ 恶化(Deterioration): MMSE curative effect index <0.
2017_Zhang_2	① 临床控制(Clinical control): the patient is clear-headed, quick-responsive, and has a sound orientation, the main symptoms have recovered, he can take care of himself, answers questions correctly, and can carry out general social activities;<br>② 显效(markedly effective): the orientation is basically sound, and the main symptoms are severe Partially recovered, can take care of themselves, answers questions are basically relevant, but the response is average;<br>③ 有效(effective): some symptoms are restored, or the main symptoms are relieved, life is basically self-care, answers to questions are basically relevant, but the response is slow, and there are still obstacles to personality and intelligence;<br>④ 无效(invalid): Symptoms did not improve after treatment, and even worsened.<br><br>The total effective rate of treatment = (clinical control + markedly effective + effective) / total number of cases × 100%
2018_Gu	NR
2020_Suns	NR
2020_Jin	① 显效(Markedly effective): BEHAVE-AD score reduced by ≥ 50%;<br>② 有效(effective): BEHAVE-AD score reduced by 25% to 49%<br>③ 无效(ineffective): BEHAVE-AD score reduced by <25%.

	Total effective rate = (marked effect + effective) / total number × 100%.
2021_Song	① 临床控制(clinical control): Behave-AD reduction ≥ 75%<br>② 显效(markedly effective): Behave-AD reduction 50–74%<br>③ 进步(progress): Behave-AD reduction 25–49%<br>④ 无效(invalid): Behave-AD reduction < 25%<br><br>Total effective rate = (clinical control + markedly effective + progress) / total number of cases 100%
2022_LI	① 显效(markedly effective): MoCA score increased ≥20%<br>② 有效(effective), MoCA score increased ≥ 12% but <20%;<br>③ 稳定(stable), MoCA score increased ≥ –12% but <12%<br>④ 恶化(deterioration), MoCA score increased <–12%.
2024_RAO	TCM syndrome score reduction rate = [(TCM syndrome score before treatment – TCM syndrome score after treatment) / TCM syndrome score before treatment] × 100%.<br><br>① 痊愈(cured): TCM score ≥95%<br>② 显效(markedly effective): 70% ≤ TCM score <95%<br>③ 好转(improvement): 30% ≤ TCM score <70%<br>④ 无效(ineffective): <30%

*footnote

Included studies are listed in References [29–40]

Behave-AD: Behavioral Pathology in Alzheimer's Disease Rating Scale; MMSE: Mini Mental State Examination; MoCA: Montreal Cognitive Assessment; TCM: Traditional Chinese Medicine

Supplementary Content S6. Forest plots for other outcomes between LWDH and mLWDH plus conventional treatment group and conventional alone group.

6.1. Cognition

(a) ADAS-cog(Alzheimer's Disease Assessment Scale-Cognitive Subscale)

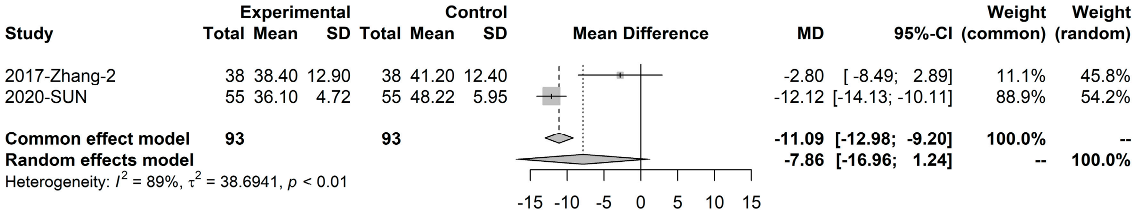

(b) ACE-R(Addenbrooke's Cognitive Examination-Revised)

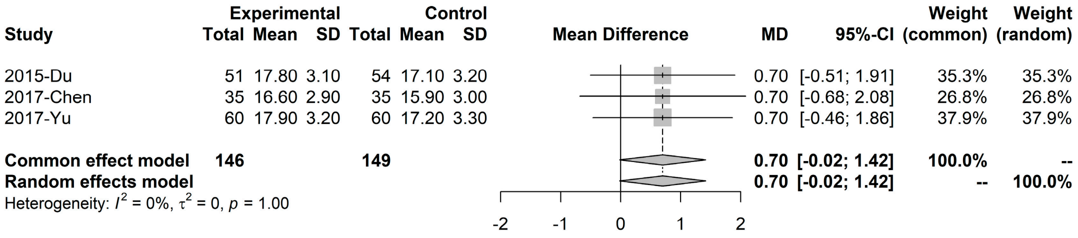

6.2. Quality of life and daily life

(a) MBI(modified Barthel Index) subgroup analysis by WM(Western Medicine)

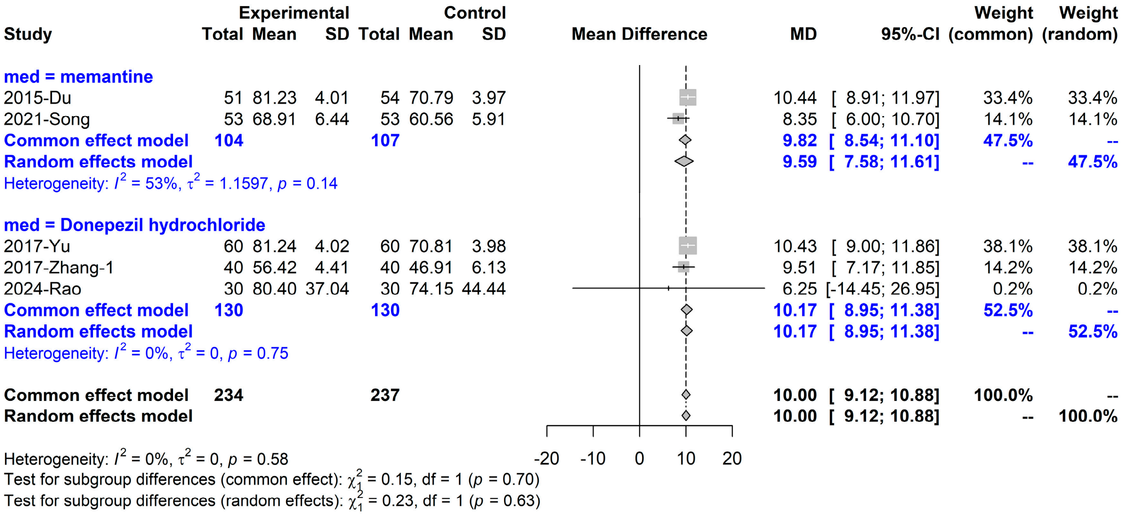

(b) ADL(Activities of Daily Living) Forest plot

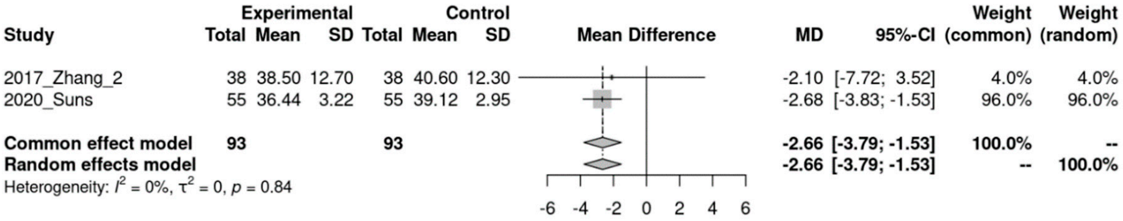

(c) QOL-AD(Quality of Life in Alzheimer's dementia scale) Forest plot

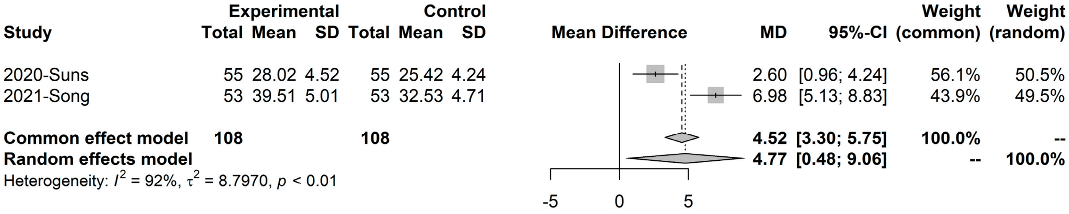

6.3. Mental status assessment

(a) BEHAVE-AD forest plots for BEHAVE-AD between the LWDH plus conventional treatment group and conventional treatment alone group

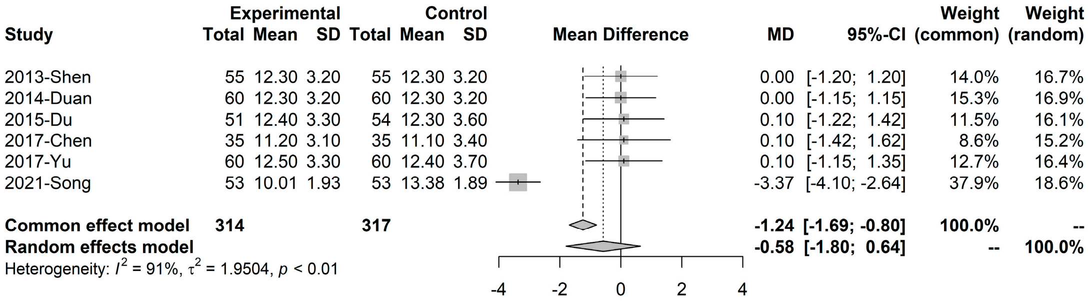

*BEHAVE-AD, Behavior Pathology in Alzhimer's Disease Rating Scale; LWDH, Liuwei Dihuang

(b) Behave-AD subgroup analysis by treatment period

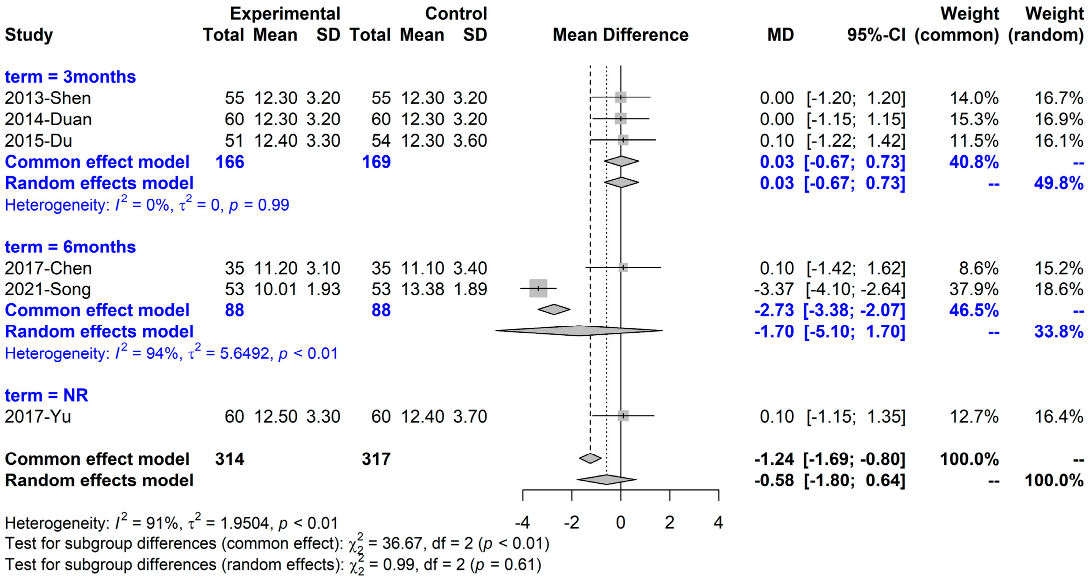

(c) BEHAVE-AD subgroup analysis using WM

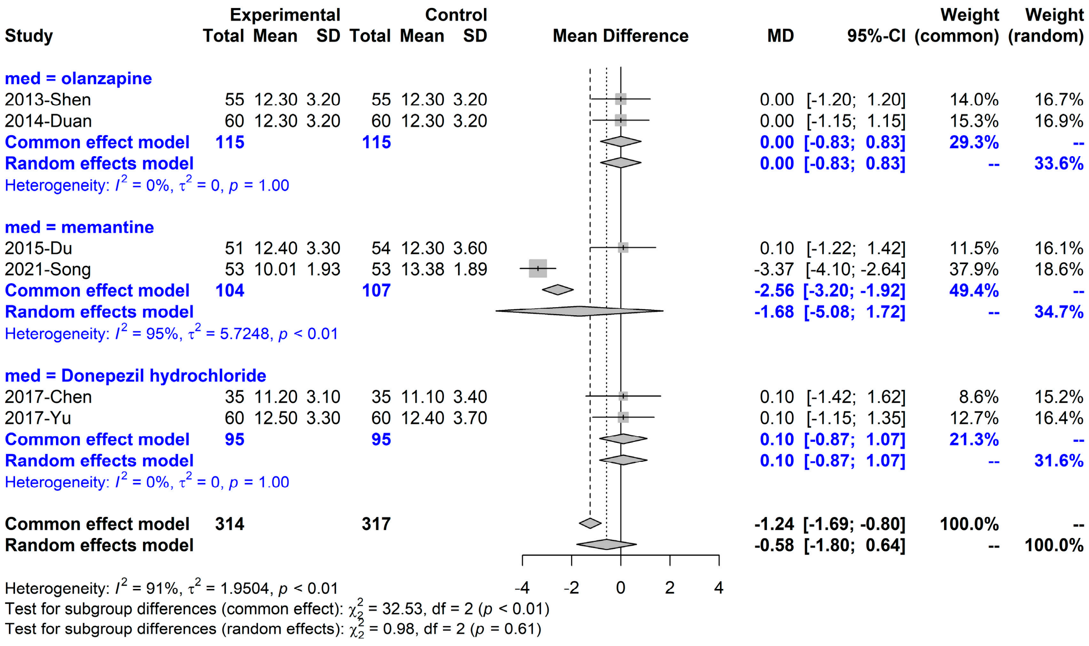

6.4. TCM(Traditional Chinese Medicine) symptoms score

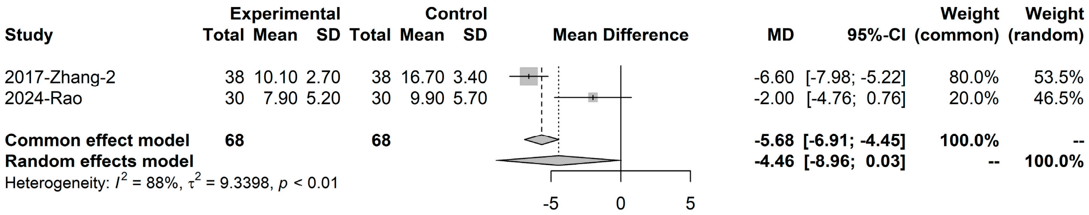

6.5. TER (Treatment effect rate) OR

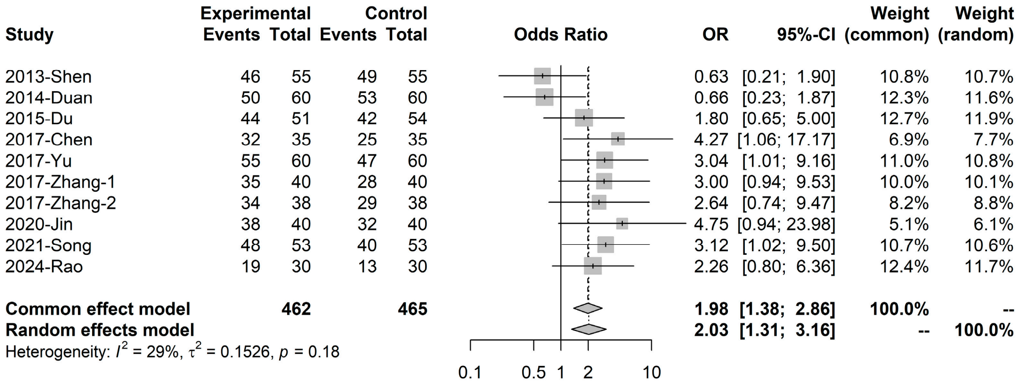

6.6. Adverse effect OR

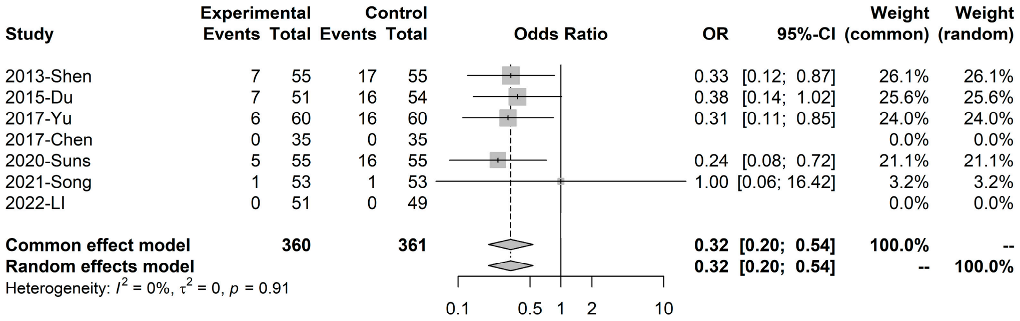

Supplementary Content S7. Forest plots of leave-one-out cross validation sorted by effect size for other outcomes

(a) MMSE(Mini-Mental State Examination) leave-one-out analysis

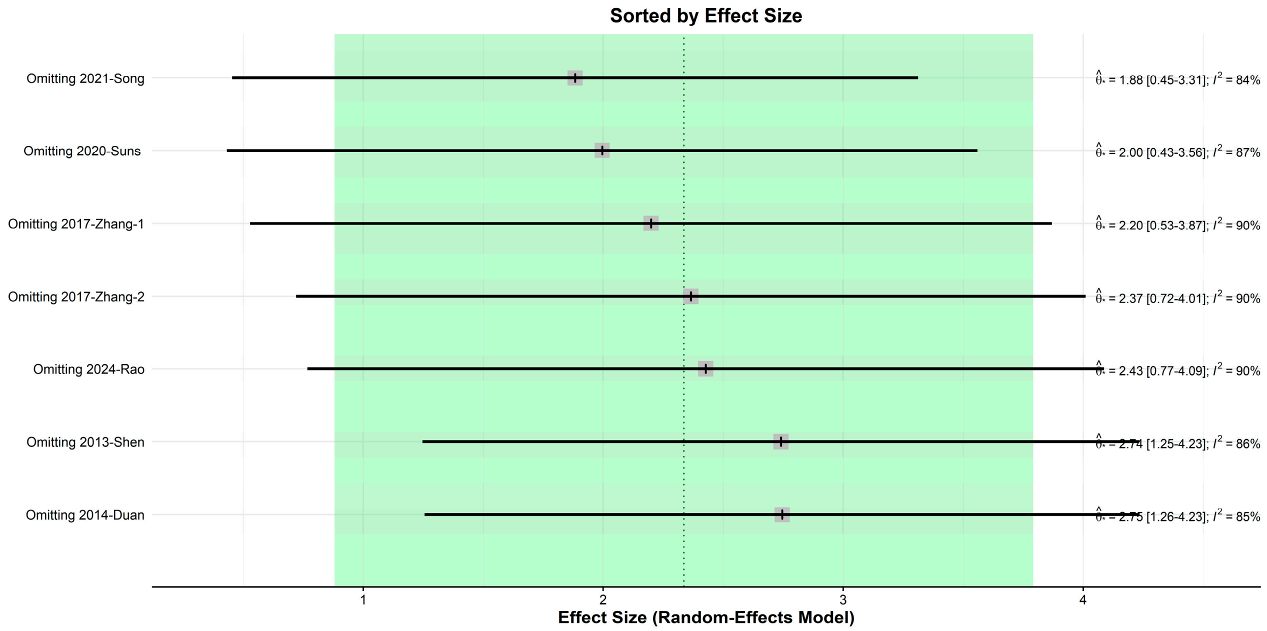

(b) MBI(modified Barthel Index) leave-one-out analysis

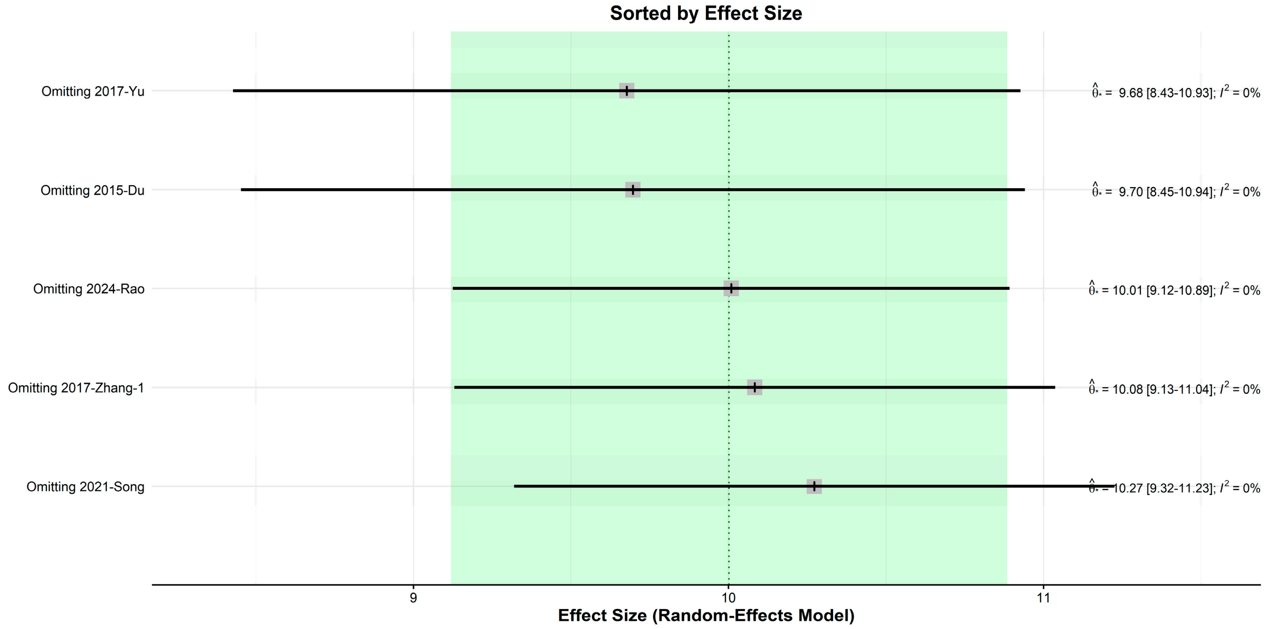

(c) Behave-AD(Behavior Pathology in Alzhimer's Disease Rating Scale) leave one-out analysis

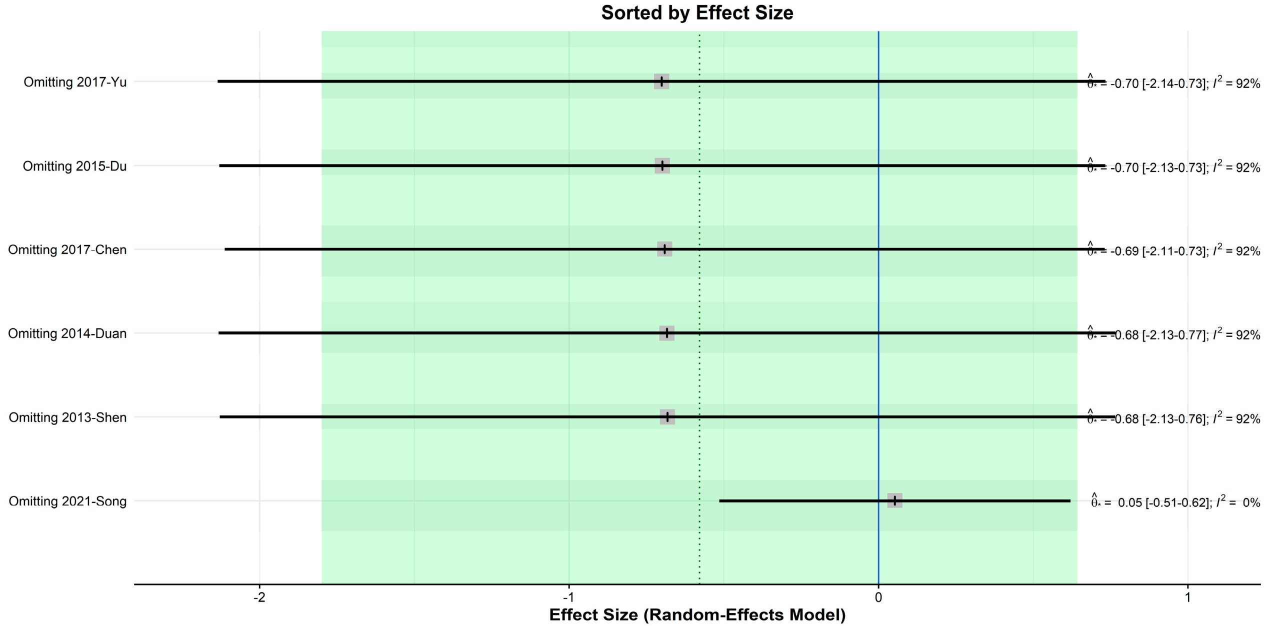

Supplementary References

(1) Fu, Y. Comparative study on the improvement effect of Liuwei Dihuang Pills and olanzapine on mental and behavioral symptoms of Alzheimer's disease. *First Peoples Hosp. Pingdingshan City Henan Prov.* **2015**, *17* (7), 55–56.

(2) Zhao, L.; Bo, Y.; Dong, G.; Zhao, Q.; Wang, Zejing; Ma, X.; Wang, Y.; Zhao, J. Effect of Liuwei Dihuang Decoction on blood pressure in patients with vascular dementia. *Chin. J. Integr. Med. Cardio-Cerebrovasc. Dis.* **2013**, *11* (1), 123–124.

(3) Zhao, D.; Wang, J.; He, B. Effects of Liuwei Dihuang Pill on Gene Polymorphism of Apolipoprotein E of Patients with Mild Cognitive Impairment. *Hosp. Tradit. Chin. Med. West. Med. Zhejiang Prov.* **2014**, *21* (1), 52–54.

(4) Zhao, L.; Bo, Y.; Zhao, Q.; Dong, G.; Wang, Z.; Ma, X.; Wang, Y.; Zhao, J. *Effects of modified Liuwei Dihuang Decoction on cerebral blood flow and blood rheology*. https://oversea-cnki-net.libproxy.wku.ac.kr/KCMS/detail/detail.aspx?dbcode=CJFD&dbname=CJFD2012&filename=ZSFX201203070&uniplatform=OVERSEA&v=RoN84WuMbiFDfeH5SIoH5cWQdGKexghYsu406jvBL2Cw7viu827mtYvEAV5m6-14 (accessed 2023-11-13).

(5) Dai, Z.; Zheng, D. 28 cases of mild cognitive impairment treated with Liuwei Dihuang Decoction and aromatic resuscitation method. *Chinas Naturop.* **2007**, No. 12, 24. https://doi.org/10.19621/j.cnki.11-3555/r.2007.12.026.

(6) Zhang, L. 30 cases of vascular dementia treated with modified Liuwei Dihuang decoction. *J. Liaoning Coll. Tradit. Chin. Med.* **2003**, No. 4, 347. https://doi.org/10.13194/j.jlunivtcm.2003.04.40.zhanglj.022.

(7) Zhang, M.; Zhang, W. 36 cases of vascular dementia treated with Liuwei Dihuang Decoction. *Shaanxi J. Tradit. Chin. Mediticne* **2001**, No. 2, 85.

(8) Ma, Y.; Gao, Y.; Wang, L.; Zhang, P.; Wang, J. Examples of clinical trials of Liuwei Dihuang Pills in treating encephalopathy. *Henan Tradit. Chin. Med.* **2008**, No. 3, 62–63. https://doi.org/10.16367/j.issn.1003-5028.2008.03.005.

(9) Li, J.; Zhou, H.; Lin, L. Observation on 40 cases of auxiliary treatment of Liuwei Dihuang Pills in delaying Alzheimer's disease. *Chin. J. Integr. Med. Cardio-Cerebrovasc. Dis.* **2014**, *12* (1), 117–118.
(10) Qing, zhao qian. Observation on the efficacy of Liuwei Dihuang Pills in delaying Alzheimer's disease in 50 cases. *Chin. J. Tradit. Med. Sci. Technol.* **2007**, No. 5, 373–374.
(11) Zhao, L.; Bo, Y.; Zhao, Q.; Dong, G.; Wang, Z; Ma, X.; Wang, Y.; Zhao, J. Observation on the efficacy of modified Liuwei Dihuang Decoction in the treatment of 40 cases of vascular dementia. *Chin. J. Tradit. Med. Sci. Technol.* **2012**, *19* (6), 550–551.
(12) Tian, L.; Zhang, S; DONG, T. On the Treatment of Alzheimer's Disease with Liu Wei Di Huang Wan from the "Jing Gua." *Chin. J. Ethnomedicine Ethnopharmacy* **2022**, *31* (15), 77-79+91.
(13) Lu, S. Summary of 33 cases of Alzheimer's disease treated with Liuwei Dihuang Decoction. *GANSU J. Tradit. Chin. Med.* **1999**, No. 2, 12.
(14) Wang, Z. Liuwei Dihuang Pills Can Treat Alzheimer's Disease. *It Help. Open Book Seek. Med. Advice* **2000**, No. 6, 46.
(15) Li, X.; Lin, R.; Yang, B.; Lu, J.; Hu, Y. Overview of Yang warming method in treating vascular dementia. *Hunan J. Tradit. Chin. Med.* **2020**, *36* (2), 145–147. https://doi.org/10.16808/j.cnki.issn1003-7705.2020.02.061.
(16) Sun, X. Overview of treating encephalopathy with Liuwei Dihuang Pills. *Shandong J. Tradit. Chin. Med.* **2009**, *28* (2), 142–143. https://doi.org/10.16295/j.cnki.0257-358x.2009.02.033.
(17) Yang, L. Observation on the therapeutic effect of heat-sensitive moxibustion combined with modified Liuwei Dihuang decoction on VaMCI with kidney essence deficiency type. 硕士, Guangzhou University of Chinese Medicine, 2018. https://chn.oversea.cnki.net/KCMS/detail/detail.aspx?dbcode=CMFD&dbname=CMFD202201&filename=1021881624.nh&uniplatform=OVERSEA&v=06DLCheQkIsgAntyE6PCdi_HUpvaypO7AKlSXjihwgDNk1gJ0bEqUuwvBXxJgFW0 (accessed 2023-11-13).
(18) Zhuang, H.; Wang, J.; Zhuang, H. effect of modified Liuwei Dihuang Decoction combined with buflodil on cognitive function and daily living ability in elderly patients with vascular dementia. *Chin. J. Integr. Med. Cardio-Cerebrovasc. Dis.* **2014**, *12* (5), 588–589.
(19) Fang, C. Clinical study on the treatment of mild to moderate vascular dementia of spleen and kidney deficiency type with modified Sangma Dihuang granules. 硕士, Shandong University of Traditional Chinese Medicine, Shandong University, 2021. https://chn.oversea.cnki.net/KCMS/detail/detail.aspx?dbcode=CMFD&dbname=CMFD202201&filename=1021129964.nh&uniplatform=OVERSEA&v=GHqDL81RWpWrvznEAXfqdOrDTV-w7oHxn08plfTi7rcIkLLUW1Zucs-TZUPYnXVV (accessed 2023-11-13).
(20) Zhang, X.; Liu, D.; Chen, X.; Zhao, J.; Bai, Y.; Jiang, Y.; Xie, R.; Lian, X. Clinical study on Liuwei Dihuang Pills combined with butylphthalide capsules in the treatment of cerebral small vessel disease accompanied by non-dementia vascular cognitive impairment. *Shanxi Med. J.* **2020**, *49* (23), 3198–3200.
(21) Zheng, Y.; Sun, M.; Tan, Y.; Zhan, G. Clinical efficacy of Qiwei Dihuang Decoction combined with alprostadil in the treatment of patients with vascular dementia and its impact on cognitive function. *Chin. Mod. Dr.* **2017**, *55* (9), 144-146+150.
(22) Chao, J.; Zhu, A. To explore the clinical effect of Liuwei Dihuang Pills in the treatment of Alzheimer's disease. *Everyones Health Acad. Ed.* **2014**, *8* (7), 39–40.
(23) Zhao, L.; Wang, Z.; Ma, X.; Wang, Y. Observation on the efficacy of modified Liuwei Dihuang Decoction in the treatment of vascular dementia with elevated high-sensitivity C-reactive protein. *Hebei Tradit. Chin. Med.* **2017**, *39* (1), 73–75.
(24) Zhang, C. Clinical study on Liuwei Dihuang Pill combined with treatment of Alzheimer's disease. *Clin. Res. Tradit. Chin. Med.* **2012**, *4* (19), 88+90.
(25) Sun, Q.; Li, F.; Tán, sòng táo. Clinical effect of Liuwei Dihuang Pills in the treatment of Alzheimer's disease and its impact on patients' quality of life. *Clin. Res. Tradit. Chin. Med.* **2017**, *9* (33), 53–54.
(26) Gu, J.; Luo, H.; Zhang, Z. To explore the effect of Liuwei Dihuang Pills and olanzapine on improving mental symptoms in patients with Alzheimer's disease. *Clin. Res. Tradit. Chin. Med.* **2018**, *10* (14), 66-67+69.

(27) Feng, J.; Zang, M. Clinical study on Liuwei Dihuang Pills in the treatment of mild to moderate vascular cognitive impairment. *Clin. J. Tradit. Chin. Med.* **2010**, *22* (2), 131–132. https://doi.org/10.16448/j.cjtcm.2010.02.034.

(28) Qing, zhao qian. Observation on the efficacy of Liuwei Dihuang Pills in delaying Alzheimer's disease in 50 cases. In Clinical Application Research of Liuwei Dihuang Preparations; Beijing Tongrentang Technology Development Co., Ltd.; Chinese Journal of Traditional Chinese Medicine, 2012; p 2.

(29) Shen, Y. Comparison of Liuwei Dihuang Pills and Olanzapine in Improving Mental and Behavioral Symptoms of Senile Dementia. *Guid. J. Tradit. Chin. Med. Pharm.* **2013**, *19* (12), 39–40. https://doi.org/10.13862/j.cnki.cn43-1446/r.2013.12.021.

(30) Duan, F.; Lu, X. Comparison of Liuwei Dihuang Pills and Olanzapine in Improving Mental and Behavioral Symptoms of Senile Dementia. *J. China Prescr. Drug* **2014**, *12* (10), 58–59.

(31) Du, G.; Li, H.; Liu, D.; Hou, Y. Application and Effect of Liuwei Dihuang Pills in Adjuvant Treatment of Alzheimer's Disease. *Hebei Med. J.* **2015**, *37* (11), 1661–1663.

(32) Yu, W. Clinical Observation on Treating Mild and Moderate Alzheimer's Disease with the Liuwei Dihuang Decoction plus Western Medicine. *Clin. J. Chin. Med.* **2017**, *9* (33), 55–57. https://doi.org/10.3969/j.issn.1674-7860.2017.33.026.

(33) Chen, J. Clinical Observation of Liuwei Dihuang Pills Combined with Donepezil Hydrochloride in the Treatment of Alzheimer's Disease of Kidney Yin Deficiency. *Chin. J. Clin. Ration. Drug Use* **2017**, *10* (1), 64–65. https://doi.org/10． 15887 /j． cnki． 13-1389 / r． 2017． 01． 036.

(34) Zhang, X. Study on Curative Effect of Combination of Traditional Chinese Medicine and Western Medicine in Treating Vascular Dementia. *Chinas Naturop.* **2017**, *25* (5), 65–66. https://doi.org/10.19621/j.cnki.11-3555/r.2017.05.056.

(35) Zhang, L.; Li, F.; Ma, L. Clinical Observation on Liuwei Dihuang Pill Combined with Western Medicine in the Treatment of Mild to Moderate Alzheimer's Disease. *World Chin. Med.* **2017**, *12* (11), 2659–2665. https://doi.org/10． 3969 /j． issn． 1673 － 7202． .

(36) Jin, H. Liuwei Dihuang Pills Combined with Donepezil Hydrochloride Observation on Clinical Curative Effect of Treating Senile Dementia of Kidney Yin Deficiency. *Electron. J. Clin. Med. Lit.* **2020**, *Vol.7* (No.3), 25,59. https://doi.org/10.16281/j.cnki.jocml.2020.03.023.

(37) SUN, S. Application Effect of Liuwei Dihuang Pill in Adjuvant Treatment of Alzheimer's Disease. *Guide China Med.* **2020**, *18* (21), 169–170. https://doi.org/10.15912/j.cnki.gocm.2020.21.080.

(38) Song, W. Effect of Liuwei Dihuang Pills Combined with Memantine on Cognitive Function and QOL-AD Score in Alzheimer's Disease. *Inn. Mong. Med. J.* **2021**, *53* (9), 1103–1105. https://doi.org/10.16096/J.cnki.nmgyxzz.2021.53.09.027.

(39) LI, B. Clinical Effects of Supplemented Liuwei Dihuang Granules Combined with Conventional Treatment on Patients with Hypertension in the Elderly Complicated with Cognitive Impairment. *Chin. Tradit. Pat. Med.* **2022**, *44* (12), 3842–3847. https://doi.org/10.3969/j.issn.1001-1528.2022.12.015.

(40) RAO, G.; PENG, Y.; ZHANG, Z.; LONG, L.; WU, S. Clinical effect of Liuwei Dihuang pills combined with donepezil hydrochloride in treatment of mild－to－moderate Alzheimer's disease: An analysis of 30 cases. *HUNAN JOU RNAL T RADITIONAL Chin. Med.* **2024**, *40* (1), 6-9,32. https://doi.org/10． 16808 /j． cnki． issn1003－7705． 2024． 01． 002.
